# Kindlin-1 Links Integrins and Maintenance of Nuclear Shape in Cutaneous Squamous Carcinoma Cells

**DOI:** 10.3390/cells15211991

**Published:** 2026-10-31

**Authors:** Alejandra Varela, Samar Sayedyahossein, Rithwik Ramachandran, Lina Dagnino

**Affiliations:** 1 Department of Physiology and Pharmacology, Western University, London, ON N6A 5C1, Canada; avarela4@uwo.ca (A.V.), samaryahossein@vt.edu (S.S.), rramach@uwo.ca (R.R.); 2 Children’s Health Research Institute, London, ON N6A 5C1, Canada; 3 Department of Oncology, Western University, London, ON N6A 5C1, Canada

**Keywords:** Kindlin-1, epidermis, cutaneous squamous cell carcinoma, nuclear morphology, F-actin cytoskeleton, RhoA GTPase

## Abstract

Kindlins are essential for integrin activation and mechanoresponses. In the epidermis, inherited loss-of-function of the *FERMT1* gene, which encodes Kindlin-1, increases the risk of developing cutaneous squamous cell carcinoma (cSCC). Notably, the role of Kindlin-1 in epidermal tumours has remained virtually unexplored. In this study, we analyzed the consequences of *FERMT1* inactivation in a cSCC cell model. Kindlin-1-depleted cells exhibited decreased number and size of focal adhesions, as well as alterations in actin stress fibres and the F-actin perinuclear cap. Significantly, *FERMT1* inactivation also resulted in reduced nuclear volume and total surface area. These abnormalities were not ameliorated by exogenous expression of Kindlin-2 or a Kindlin-1 mutant incapable of binding to integrins. In contrast, nuclear dimensions in Kindlin-1-deficient cells were restored in the presence of a constitutively active RhoA protein. These findings are consistent with the concept that Kindlin-1 plays an essential role mediating integrin signalling and RhoA activation to modulate perinuclear actin filaments and nuclear morphology.

## 1. Introduction

Kindlins are adaptor proteins that are recruited to and activate integrins at early stages of focal adhesion assembly [1]. As essential components of the integrin adhesome, kindlins not only serve as scaffolds, but also as signalling hubs [2]. In this manner, kindlins coordinate integrin function, F-actin cytoskeletal dynamics, cell adhesion, migration and mechanics. 

The three known mammalian kindlin forms share about 60% homology, exhibit distinct expression patterns and fulfill overlapping, but not identical, functions [3]. Kindlin-1 is expressed in the epidermis and other epithelial tissues, Kindlin-2 is ubiquitous, and Kindlin-3 is found in osteoclasts, endothelial and hemopoietic cells [3]. In epidermal keratinocytes, Kindlin-1 binds to β1 and β6 integrins, and modulates epidermal growth factor receptor signalling and endosomal recycling, as well as maintenance of redox balance and adhesion to the extracellular matrix (ECM) [4,5,6,7]. Inherited loss-of function mutations in *FERMT1*, which encodes Kindlin-1, give rise to epidermal fragility and blistering, photosensitivity, atrophy, chronic cutaneous inflammation and elevated incidence of cutaneous squamous cell carcinoma (cSCC) [8].

Kindlins are necessary to activate integrins. The effect of integrin activation by ECM substrates and/or mechanical forces extends well beyond focal adhesion assembly and F-actin remodelling. In particular, a highly organized perinuclear network of actin filaments, termed the actin cap, is physically connected to the nuclear envelope through interactions with the Linker of Nucleoskeleton and Cytoskeleton (LINC) complex [9]. Such interactions establish bidirectional force transmission and activate signalling pathways between the extracellular environment and the nucleus. These processes result in changes in nuclear morphology, chromatin accessibility and activation of transcriptional responses with important physiological and pathological outcomes [9,10]. Indeed, environmental mechanics, as well as nuclear stiffness and morphology, contribute to cell lineage specification and maintenance of genomic integrity, but also to maladaptive chromatin remodeling. The latter can lead to profibrotic gene activation, genomic instability and activation of oncogenic transcriptional programs [11,12].

Cutaneous SCC is one of the most common cancers with high metastatic potential. UV irradiation, immunosuppression and chronic inflammation are major etiological factors in cSCC. In individuals with genetic blistering disorders, chronic inflammation causes abnormalities in the ECM, fibrosis, tissue stiffening and abnormal mechanosignalling via integrins [13]. Sporadic cSCC neoplasms also exhibit elevated stiffness relative to adjacent normal tissues, facilitating tumour cell proliferation and tissue invasion [14,15]. Although the role of Kindlin-1 in non-transformed epidermal keratinocytes has been extensively studied, its functions in cSCC remain virtually unexplored. To begin to address this gap, we have investigated the consequences of *FERMT1* inactivation on the actin cytoskeleton and nuclear morphology in human cSCC cells. Our findings indicate an essential role for Kindlin-1 in the modulation of focal adhesions, the F-actin cytoskeleton and nuclear dimensions in these cells. We further demonstrate that this role requires the ability of Kindlin-1 to bind to integrin cytoplasmic tails, and that Kindlin-2 is unable to fully substitute Kindlin-1 in the modulation of focal adhesions and nuclear dimensions in these cells.

## 2. Materials and Methods

### 2.1. Cell Culture and Transient Transfections

The SCC-13 cell line was derived from a facial epidermal squamous cell carcinoma in a 56-year-old female who received neoadjuvant radiation therapy before surgery [16]. The cells were a kind gift from Dr. Fiona Watt (King’s College London, London, UK).

Kindlin-1-deficient SCC13 monoclonal cell lines were generated by CRISPR/Cas9-mediated editing of the *FERMT1* gene in SCC13 cells. To this end, parental SCC-13 cells were transiently transfected with a - pSpCas9n(BB)-2A-GFP based vector (Addgene 48138) [17], modified to encode two gRNAs that targeted Exon 2 of the *FERMT1* gene. The gRNA sequences are: 5′-CACCGACGTCACACTGAGAGTATC-3′ and 5′-AAACGATACTCTCAGTGTGACGTC-3′. Transfected cells were isolated by fluorescence-activated cell sorting, based on GFP fluorescence, and single cells were subsequently expanded as monoclonal populations. To identify candidate clonal lines for further analysis, genomic DNA was isolated and the presence of indels (indicative of *FERMT1* editing) was assessed [17] with T7 Endonuclease I EnGen^®^ Mutation Detection kits (New England Biolabs, Ipswitch, MA, USA; NEB E3321), following the manufacturer’s instructions, and using the following primers: 5′-ATATCTGGAGCACCTGGAACC-3′ and 5′-CACCAGCCATTAGTGGTGAGT-3′. Absence of detectable Kindlin-1 protein was subsequently verified by immunoblot analysis. Additional details on the generation of these monoclonal cell lines will be described elsewhere.

Immortalized mouse dermal fibroblasts (IMDFs) are a spontaneously immortalized cell line isolated from the dermis of 2-day-old CD-1 mice [18]. All cells were cultured in Dulbecco’s Modified Eagle Medium (DMEM; Wisent Bioproducts, Saint-Jean-Baptiste, QC, Canada, 319-005-CL) containing 8% fetal bovine serum (FBS; Life Technologies, Carlsbad, CA, USA, 12483-020) and verified to be free of mycoplasma contamination (quarterly test intervals) using MycoAlert^®^ Plus Mycoplasma Detection Kits (Lonza, Walkersville, MD, USA; LT07-710). All experiments were conducted with cells that had been passaged for less than 15 generations.

For transient transfections, SCC-13 cells were seeded onto uncoated 8-well µ-Slides (ibidi, Gräfelfing, Germany, 8006) at a density of 4 × 10^4^ cells/cm^2^ and cultured overnight to yield ~70% confluent cultures prior to transfection. The cells were transfected using Lipofectamine™ 3000 (Thermo Fisher Scientific, Waltham, MA, USA, L3000015), according to the manufacturer’s protocol, using 0.5 µg DNA/cm^2^ of culture surface. Cells were cultured with DNA-lipofectamine complexes for 8 h, followed by a culture medium change and 28 additional hours prior to processing for immunofluorescence microscopy. IMDF cells were transfected using polyethyleneimine (PEI, Linear MW 25,000, Polysciences Inc., Warrington, PA, USA, 23966-100), as described [19].

### 2.2. Reagents, Vectors and Antibodies

The vector pSpCas9n(BB)-2A-GFP (px451) was a gift from Feng Zhang (Addgene plasmid # 48138; http://n2t.net/addgene:48138; RRID:Addgene_48138) [17]. Vectors encoding FLAG-tagged wild-type (wt) and mutant Kindlin proteins were a kind gift from Dr. David A. Calderwood (Yale University School of Medicine, New Haven, CT, USA) [20]. Plasmids encoding GFP and GFP-tagged RhoA T19N were purchased, respectively, from Clontech (Palo Alto, CA, USA, 6084-1) and Addgene (Watertown, MA, USA, 12967; deposited as a gift from Dr. Gary Bokoch [21]). Vectors encoding GFP-tagged wt RhoA, RhoA G14V, Cdc42 G12V and Rac1 G12V were generous gifts from Dr. Tzuu-Shuh Jou (National Taiwan University, Taipei, Taiwan). Antibodies, fluorescence reagents, their sources and application details are listed in Supplemental Table S1.

### 2.3. Cell Lysates and Immunoblot Analysis

Cell lysates were prepared with a modified RIPA buffer, as described [22,23]. Protein concentrations in the lysates were determined using Bradford assays (Bio-Rad Protein Assay Dye Reagent Concentrate, Bio-Rad Laboratories, Hercules, CA 500-0006, USA), following the manufacturer’s instructions. Proteins in lysates were resolved by denaturing gel electrophoresis, transferred to 0.2-μm polyvinylidene fluoride membranes, and probed with antibodies indicated in individual experiments.

### 2.4. Immunofluorescence and Confocal Microscopy

Cells were processed for immunofluorescence microscopy as described [24]. To stain F-actin, cells were incubated with Alexa Fluor^TM^ -conjugated phalloidin (100 µL/cm^2^; 15 min; Supplemental Table S1) and Hoechst 33342 (20 μg/mL; Thermo Fisher Scientific, H1399). Specimens on μ-Slides were mounted with 100 μl/well Epredia™ Immu-Mount™ mounting medium (Thermo Fisher, 9990402). Micrographs were obtained with a Leica DMIRBE microscope (Leica Microsystems, Wetzlar, Germany) equipped with an ORCA-ER digital camera (Hamamatsu Photonics, Hamamatsu, Japan) and Volocity 7.0.0 imaging software (Improvision, Coventry, UK). Confocal microscopy images were obtained using a Nikon ECLIPSE Ti2-E inverted microscope (Nikon Corporation, Tokyo, Japan) with a X-Light V2 L-FOV Spinning Disk System (CrestOptics S.p.A., Rome, Italy), an ORCA-Fusion digital camera (Hamamatsu Photonics, Hamamatsu, Japan) and NIS-Elements AR imaging software 5.30.06 (Nikon Instruments Inc., New York, NY, USA). Z-stacks were acquired with a 60X/1.4NA oil objective, with a step size of 0.3 µm and laser power set to 10%. 

### 2.5. Analysis of Focal Adhesions

Focal adhesion numbers and area were quantified based on phospho-paxillin immunoreactivity, using Fiji v2.16.0 [25] as described [26], with the following modifications. Prior to analysis, images were screened to determine if they met predefined criteria based on background signal intensity. Images with a background mean grey value above 10 pixels were excluded. Compliant images were processed using a custom Fiji macro to automate background subtraction, local contrast enhancement, signal exponentiation and thresholding steps. An additional processing step, watershed, was incorporated to allow clear resolution of focal adhesions. 

### 2.6. Analysis of Nuclear Morphology

Nuclear volume and total surface area were analyzed from confocal z-stack images of cells labelled with anti-lamin A/C antibodies, using the NucleusJ 2.0 Plugin for Fiji [27]. Nuclei that overlapped in micrographs were excluded from the analysis. The segmented z-stacks generated with the NucleusJ 2.0 Plugin were also converted into 2D nuclear masks for circularity measurements. Circularity values were calculated from maximum intensity projection images, using the following equation: Circularity = 4πA/P^2^, where A = nuclear area and P = nuclear perimeter.

### 2.7. Analysis of Supranuclear F-Actin

To quantify fluorescence associated with F-actin bundles localized to the apical aspect of the nucleus (hereafter termed “Supranuclear F-acti”), we developed a custom image analysis pipeline to be used with Fiji. For this analysis, we used multi-channel confocal z-stacks of nuclei labelled with anti-lamin A/C antibodies and Alexa Fluor^®^ 488-conjugated phalloidin, to visualize F-actin. The lamin A/C channel was used to: (1) define the nuclei to be analyzed; (2) generate nuclear masks; and (3) automatically segment the z-stacks to identify the upper boundary of the nuclei along the *z*-axis, as defined by lamin A/C immunoreactivity. The F-actin channel was used to generate a 2-dimensional maximum intensity projection of the z-stacks located above the upper boundary of the nuclei. In addition, we also generated 2-dimensional maximum intensity projections of the nuclear lamin A/C signal, which we used to calculate total nuclear area. The supranuclear F-actin projection was processed after background correction and segmented using the Otsu method. The fraction of 2-dimensional area occupied by supranuclear F-actin (i.e., phalloidin fluorescence) relative to the 2-dimensional total nuclear area was then determined. 

### 2.8. Statistical Analysis

Statistical analyses were conducted with GraphPad Prism v8.0.2, using a mixed-effects model within a repeated measures Two-way ANOVA with Tukey’s post hoc test. The Prism software uses the term “repeated measures” for randomized block analysis. Significance was set at *p* < 0.05. Results shown are representative of at least three biological replicates, scoring 10–45 cells per replicate, as indicated in individual experiments.

## 3. Results

### 3.1. Functional Inactivation of FERMT1 Alters Focal Adhesions in SCC13 Cells

**Figure 1:**
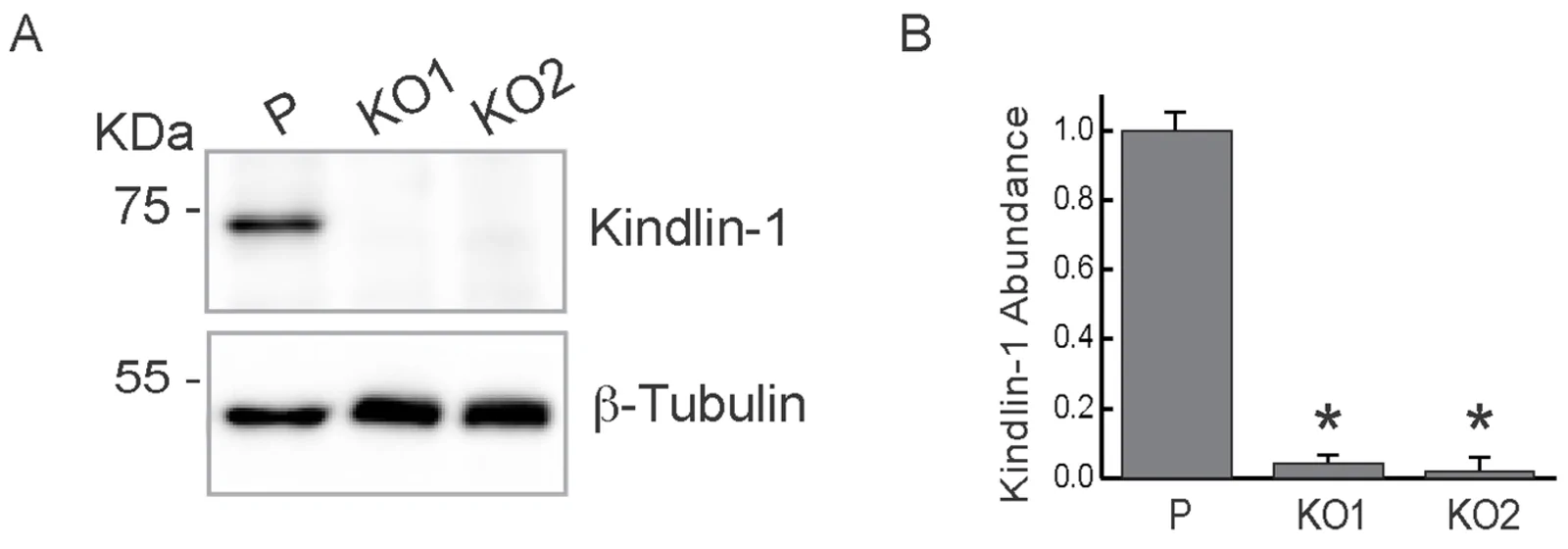
Kindlin-1 expression in SCC13 cell lines. Analysis of lysates from parental (P) SCC13 cells, or Kindlin-1-deficient SCC13 clones derived from parental cells (KO1, KO2). (**A**) Representative immunoblot probed with antibodies against Kindlin-1 and β-tubulin, used as loading control. (**B**) Densitrometric quantification of Kindlin-1 levels normalized to the corresponding β-tubulin levels. The histogram shows the mean + SD; *, *p* < 0.0001, relative to parental cells (one-way ANOVA with Tukey’s post hoc test, n = 5).

Kindlins bind to β integrin cytoplasmic tails at domains proximal to the plasma membrane. This interaction promotes integrin binding to extracellular matrix substrates, focal adhesion formation and maintenance, as well as transmission of mechanical signals [1,28]. To investigate the involvement of Kindlin-1 mechanotransduction in transformed epidermal cells, we generated two distinct Kindlin-1-deficient monoclonal populations derived from SCC13 squamous carcinoma cells (hereafter termed KO1 and KO2 cells) via CRISPR-mediated gene editing of the *FERMT1* gene, which encodes Kindlin-1 (Figure 1). 

**Figure 2:**
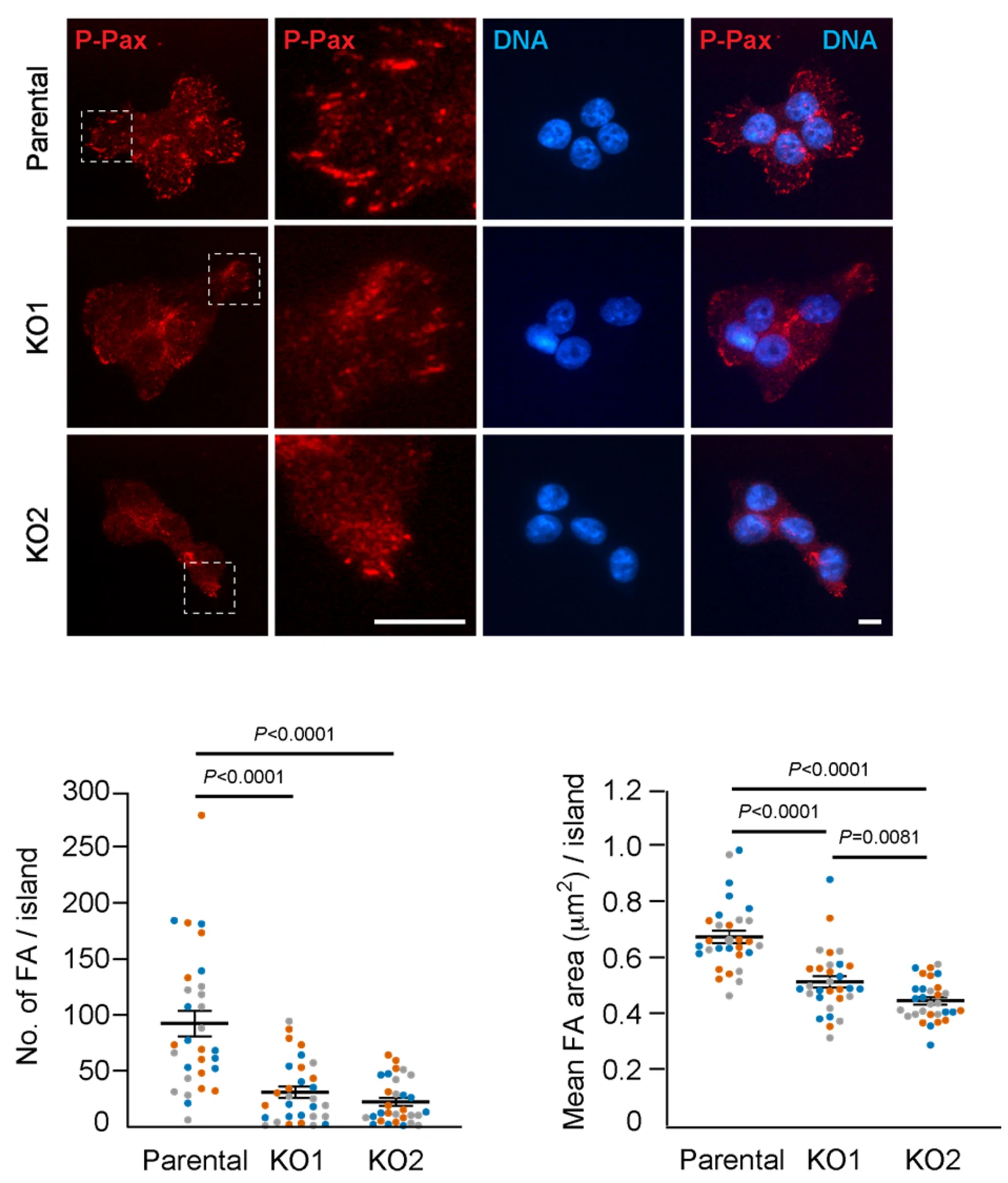
Effect of *FERMT1* inactivation on focal adhesions. Cultures of the indicated cell lines were processed for immunofluorescence microscopy with anti-phospho-paxillin (P-Pax) antibodies and Hoechst 33342 to visualize, respectively, focal adhesions and nuclear DNA. Boxed areas are shown at higher magnification in the micrograph at right. Scale bar, 10 μm. Graphs indicate the number of focal adhesions and the mean area of individual focal adhesions per cluster of 4 cells. The data represent mean ± SEM (mixed-effects model with Tukey’s post hoc test, n = 3; 10 clusters of four cells each per biological replicate. Grey, blue and orange data points represent values from a given individual biological replicate.

We first investigated whether depletion of Kindlin-1 alters focal adhesions in these cells. Because SCC13 cells grow forming clusters composed of several cells, we measured the number and surface area of focal adhesions in islands of four cells. This approach allowed clear resolution of focal adhesions, shown through phospho-paxillin immunoreactivity. The number of focal adhesions per cluster in parental cells was 92 ± 11.3, about 3- to 4-fold greater than that in KO1 and KO2 cells (31 ± 5.06 and 22.2 ± 3.59, respectively; Figure 2). Similar differences between parental and KO cells were observed when focal adhesion abundance was normalized to cluster surface area (Supplemental Figure S1). Further, the surface area of individual focal adhesions in parental cells (0.677 ± 0.022 µm^2^) was significantly decreased in KO1 and KO2 cells (0.516 ± 0.021 µm^2^ and 0.448 ± 0.013 µm^2^, respectively; Figure 2). 

**Figure 3:**
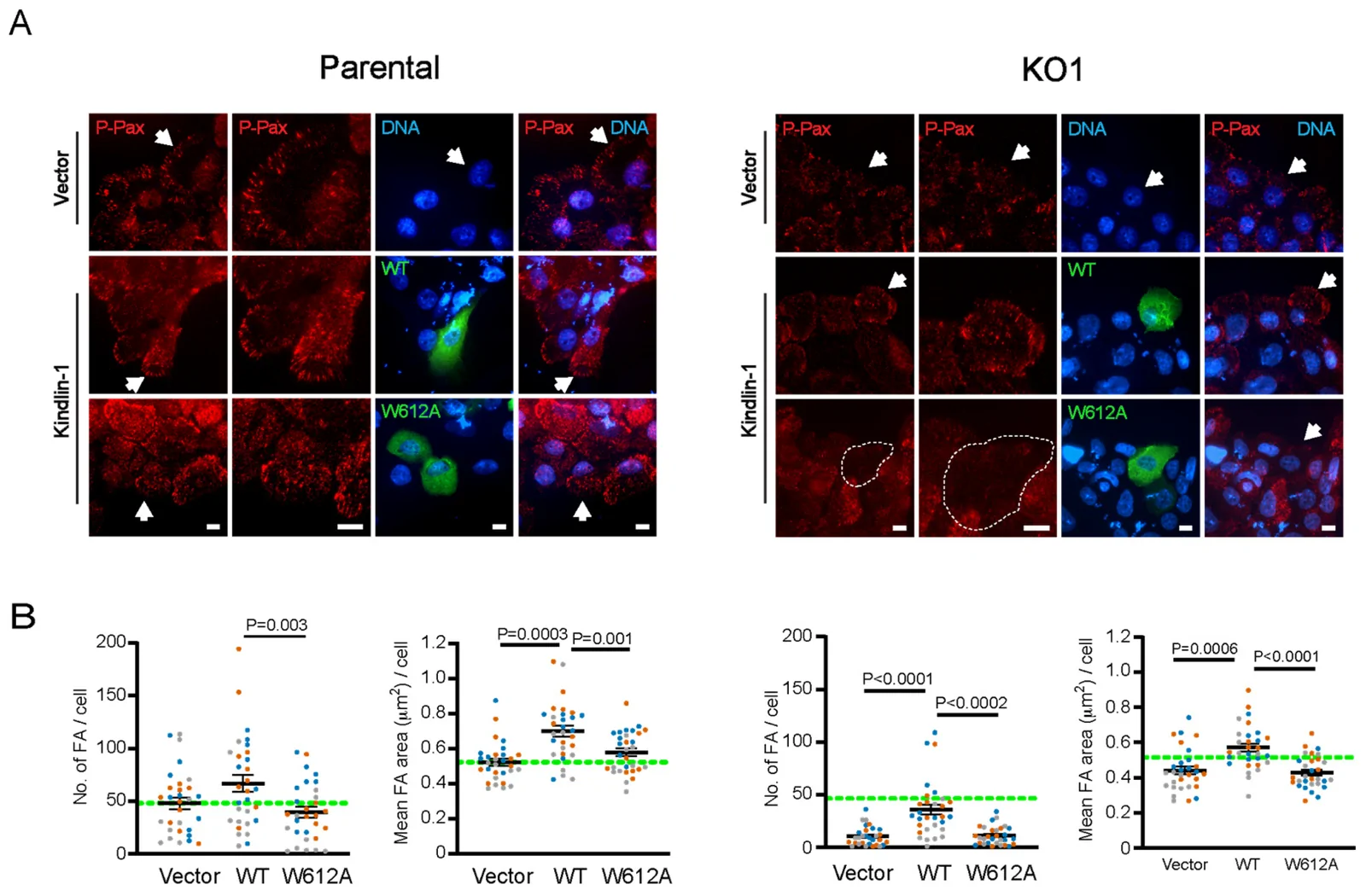
Effect of Kindlin-1 proteins on focal adhesions in SCC13 cells. (**A**) The indicated cell lines were transfected with vectors encoding FLAG-tagged wild type (WT) or W612A Kindlin-1. Twenty-eight hours after transfection, the cells were processed for immunofluorescence microscopy, using anti-phospho-paxillin (P-Pax) antibodies and Hoechst 33342 to visualize, respectively, focal adhesions and nuclear DNA. Arrows and dashed outline show transfected cells. Scale bar, 10 μm. (**B**) The graphs show values for individual cells, and bars represent mean ± SEM. Green dashed lines indicate the mean value of SCC13 parental cells transfected with control vector (mixed-effects model with Tukey’s post hoc test, n = 3, ten cells scored per experiment). Grey, blue and orange data points represent values from a given individual biological replicate.

Next, we determined the effect of exogenously expressing FLAG-tagged wild-type (WT) Kindlin-1 on focal adhesion numbers and surface area. In parental cells, exogenous expression WT Kindlin-1 did not affect the number of focal adhesions per cell. In KO1 and KO2 cells, expression of WT Kindlin-1 restored the number of focal adhesions to similar levels to those measured in parental cells transfected with control vector (Figure 3, Supplemental Figure S2; Supplemental Table S2). Of note, the surface area of individual focal adhesions in parental cells expressing WT Kindlin-1 was significantly greater than those transfected with control vector. In KO cells, expression of WT Kindlin-1 restored focal adhesion surface area to values comparable to those of parental cells transfected with the control vector (Figure 3, Supplemental Figure S2; Supplemental Table S2). These effects were dependent on the ability of Kindlin-1 to bind to integrin cytoplasmic tails, as expression of an integrin binding-deficient Kindlin-1 mutant form (W612A) [20] failed to restore focal adhesion abundance and surface area in KO1 and KO2 cells, but was without effect when expressed in parental cells (Figure 3, Supplemental Figure S2, Supplemental Table S2). 

**Figure 4:**
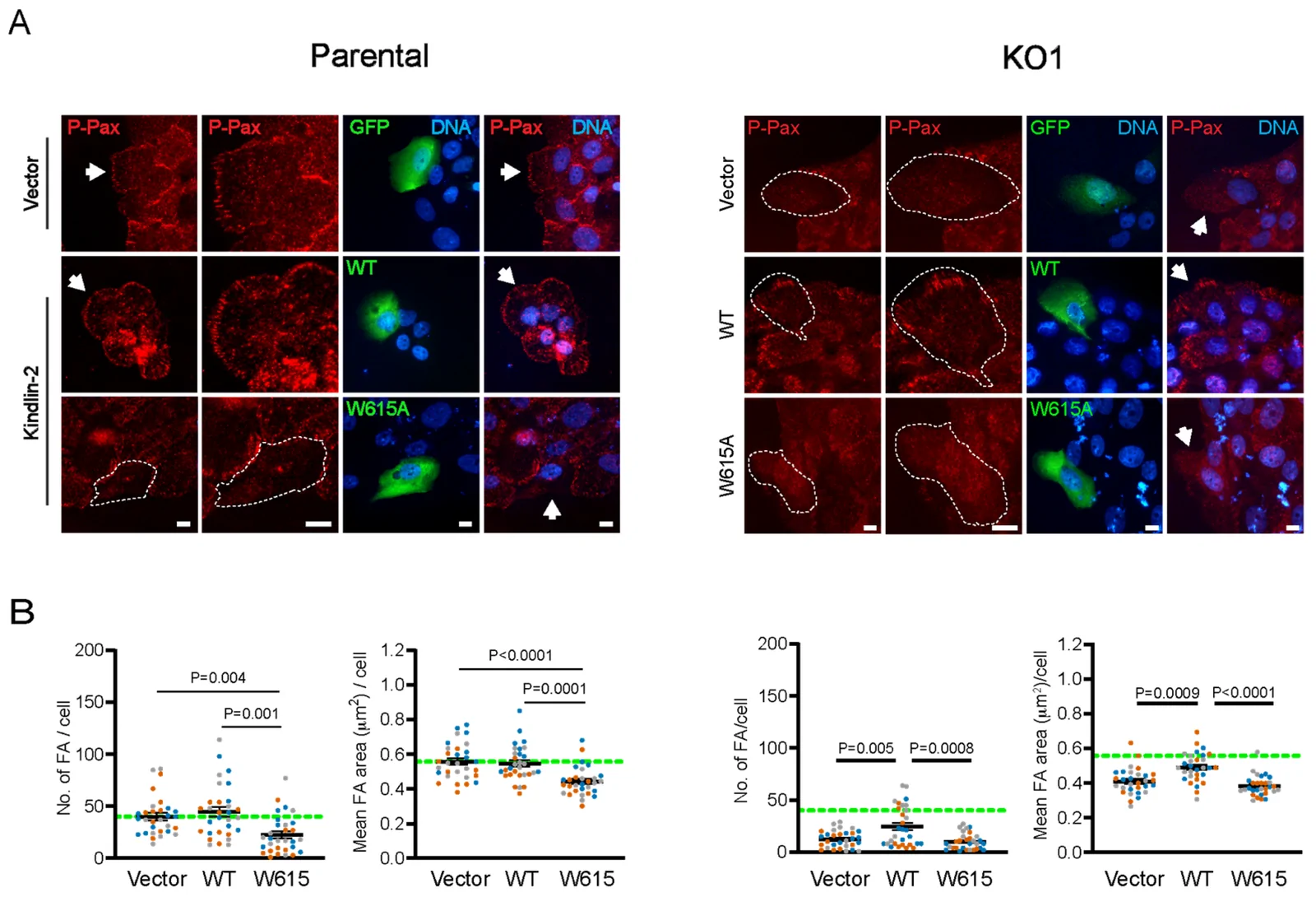
Effect of Kindlin-2 proteins on focal adhesions in SCC13 cells. (**A**) The indicated cell lines were transfected with vectors encoding control GFP, FLAG-tagged wild type (WT) or W615A Kindlin-2. Twenty-eight hours after transfection, the cells were processed for immunofluorescence microscopy, using anti-phospho-paxillin antibodies and Hoechst 33342 to visualize, respectively, focal adhesions and nuclear DNA. Arrows and dashed lines show transfected cells. Scale bar, 10 μm. (**B**) The graphs show values for individual cells, and bars represent mean ± SEM. Green dashed lines indicate the mean value of parental cells transfected with control vector (mixed-effects model with Tukey’s post hoc test, n = 3, 10 cells scored per experiment). Grey, blue and orange data points represent values from a given individual biological replicate.

Kindlin-1 and -2 exhibit unique, but also overlapping, functions in epithelial cells [4,5]. Thus, we next investigated whether Kindlin-2 might mitigate the observed abnormalities in focal adhesions in KO cells. Exogenous expression of FLAG-tagged WT Kindlin-2 in parental cells did not alter the number or the area of focal adhesions, relative to cells transfected with control vector. Similar experiments in KO cell lines showed that WT Kindlin-2 could only partially restore focal adhesion abundance and surface area, despite being present at comparable levels to those of exogenous Kindlin-1 proteins, which were previously shown to fully restore focal adhesions in KO cells (Figure 4; Supplemental Figures S3 and S4; Supplemental Table S3). 

Notably, exogenous expression of a FLAG-tagged Kindlin-2 mutant unable to bind to integrins (K2-W615A) [20] resulted in a significant decrease in the number and area of focal adhesions in parental cells (Figure 4; Supplemental Table S3). In KO cells, expression of K2-W615A did not further decrease either focal adhesion abundance or surface area, compared to vector-transfected KO cultures (Figure 4, Supplemental Figure S4; Supplemental Table S3). Collectively, these observations indicate that the ability of Kindlin-1 to effectively modulate focal adhesions in SCC13 cells requires its association with β integrin cytoplasmic tails, and that Kindlin-2 is unable to fully replace Kindlin-1 function with respect to focal adhesion assembly and/or maintenance in these cells.

### 3.2. Alterations in F-Actin and Microtubule Networks in Kindlin-1-Deficient SCC13 Cells

**Figure 5:**
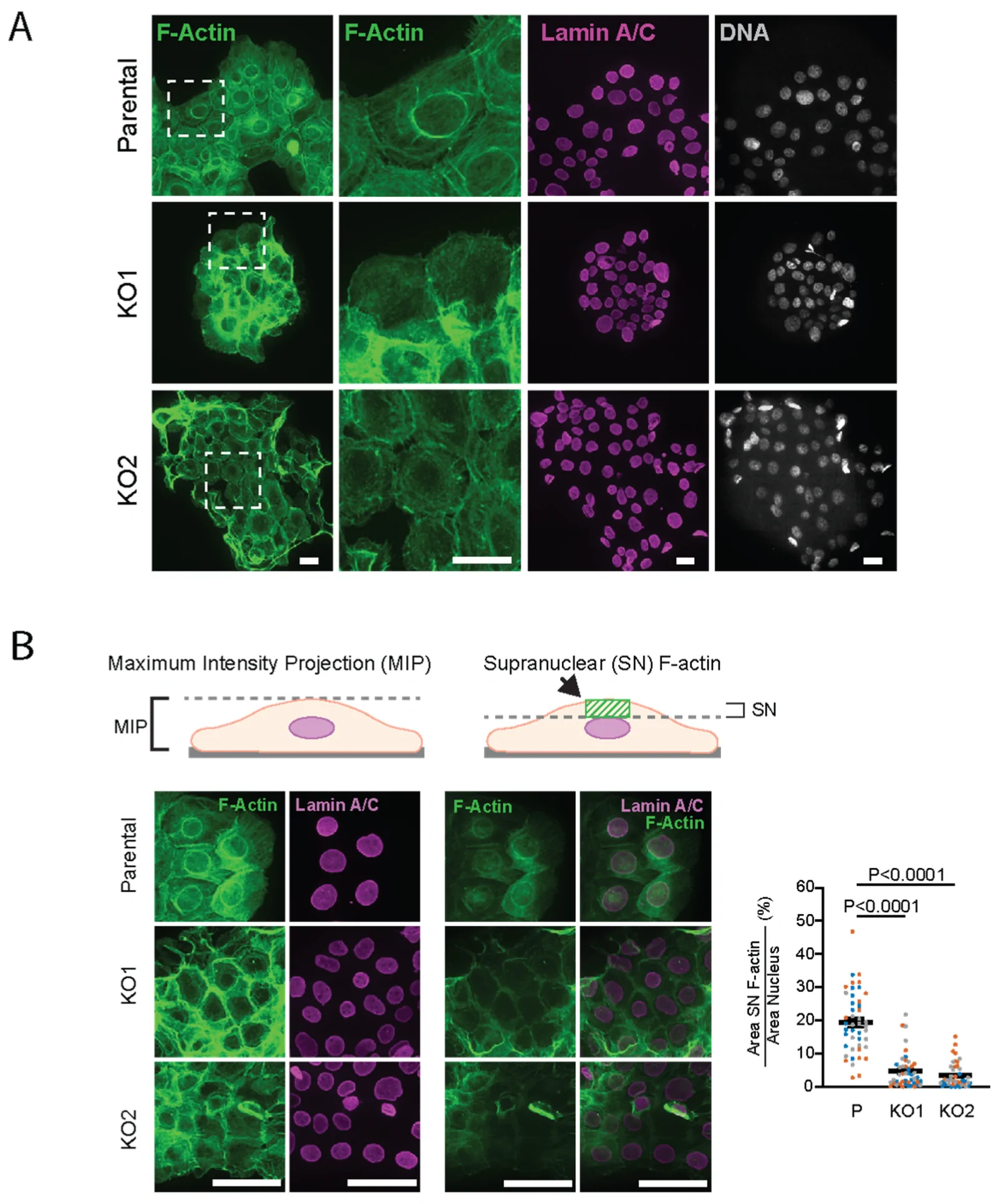
Alterations in F-actin in SCC13 KO cells. (**A**) Confocal micrograph 2-dimensional maximum intensity projection images of the indicated cell lines processed with Alexa 488-conjugated phalloidin, anti-lamin A/C antibodies and Hoechst 33342 to visualize, respectively, F-actin, the nuclear envelope and nuclear DNA. Boxed areas are shown at higher magnification in the micrograph at right. Scale bar, 25 μm. (**B**) Confocal micrograph 2-dimensional maximum intensity projections of the indicated cell lines processed with Alexa 488-conjugated phalloidin and anti-lamin A/C antibodies to visualize, respectively, F-actin and the nuclear envelope. Scale bar, 50 μm. The schematics indicate the total cellular F-actin signal from maximum intensity projections (at **left**) or F-actin fluorescence from supranuclear regions, represented by the hashed box (SN, at **right**). The graph shows the area of supranuclear (SN) F-actin fluorescence as a percent of nuclear surface area from 2-dimensional maximum intensity projections (mean ± SEM; mixed-effects model with Tukey’s post hoc test, n = 3, 15 cells scored per experiment). Grey, blue and orange data points represent values from a given individual biological replicate.

Focal adhesions connect the ECM with the actin cytoskeleton, modulating actin dynamics [29]. The focal adhesion abnormalities we observed in KO SCC13 cells prompted us to examine whether these cells also exhibit alterations in F-actin organization. In parental cells, stress fibres spanning the cell body and peripheral F-actin bundles parallel to the plasma membrane were visible, as shown by phalloidin-associated fluorescence. A perinuclear F-actin ring was also readily observed. In contrast, stress fibres were undetectable in the majority of KO1 and KO2 cells, and perinuclear F-actin rings were substantially less prominent or absent (Figure 5A).

Present in cells is also a subset of actin filaments, distinct from the stress fibres, which consists of tightly organized F-actin bundles above the apical surface of the nucleus, forming the actin cap [30]. The actin cap associates with the nuclear envelope, via LINC complexes and also with focal adhesions at the cell periphery, which are under higher tension than those connected with F-actin stress fibres. This arrangement makes the nuclear actin cap a key transducer of mechanoresponses [30]. Whereas in parental cells examination by confocal microscopy readily revealed a network of actin fibres located throughout the apical aspect of the nucleus, those networks appeared absent or substantially reduced in KO1 and KO2 cells (Figure 5B). 

To better understand these differences, we measured the area occupied by phalloidin-associated fluorescence specifically in the region directly overlaying the nucleus. We reasoned that this would provide additional information regarding alterations associated with all actin filaments apical to the nuclear plane, including the perinuclear actin cap, as well as any cortical F-actin in that region [31,32]. Hereafter, we collectively refer to these fibres as “Supranuclear F-actin”. For this analysis, we expressed “Supranuclear F-actin” fluorescence as a percentage of the nuclear surface area obtained from maximum intensity projections, as shown in the schematics of Figure 5B. Whereas the “Supranuclear F-actin”-associated fluorescence area constituted 19.3% ± 1.4% of total nuclear area in parental cells, in KO1 and KO2 cells it was only 4.6% ± 0.77% and 3.3% ± 0.55%, respectively (Figure 5B). Altogether, these observations are consistent with the notion that Kindlin-1 plays key roles in the assembly and/or maintenance of F-actin stress fibres, as well as actin filaments overlaying the nucleus, likely including the perinuclear actin cap.

Focal adhesions are physically connected with microtubule tips via KANK proteins. In this manner, microtubules can be targeted to focal adhesions, effectively resulting in crosstalk between the actin cytoskeleton and the microtubule network [29,33]. Given the observed defects in focal adhesions and F-actin in KO1 and KO2 cells, we examined if microtubules are also altered in the absence of Kindlin-1. Of note, microtubules in KO1 and KO2 cells displayed a disorganized, criss-cross or tangled pattern, compared to parental cells. Individual microtubules appeared less aligned and often bent at regions adjacent to the plasma membrane (Supplemental Figure S5). Thus, Kindlin-1 may also be important for microtubule capture by integrin-containing focal adhesions.

### 3.3. Alterations in Nuclear Morphology and the Nuclear Envelope in the Absence of Kindlin-1

**Figure 6:**
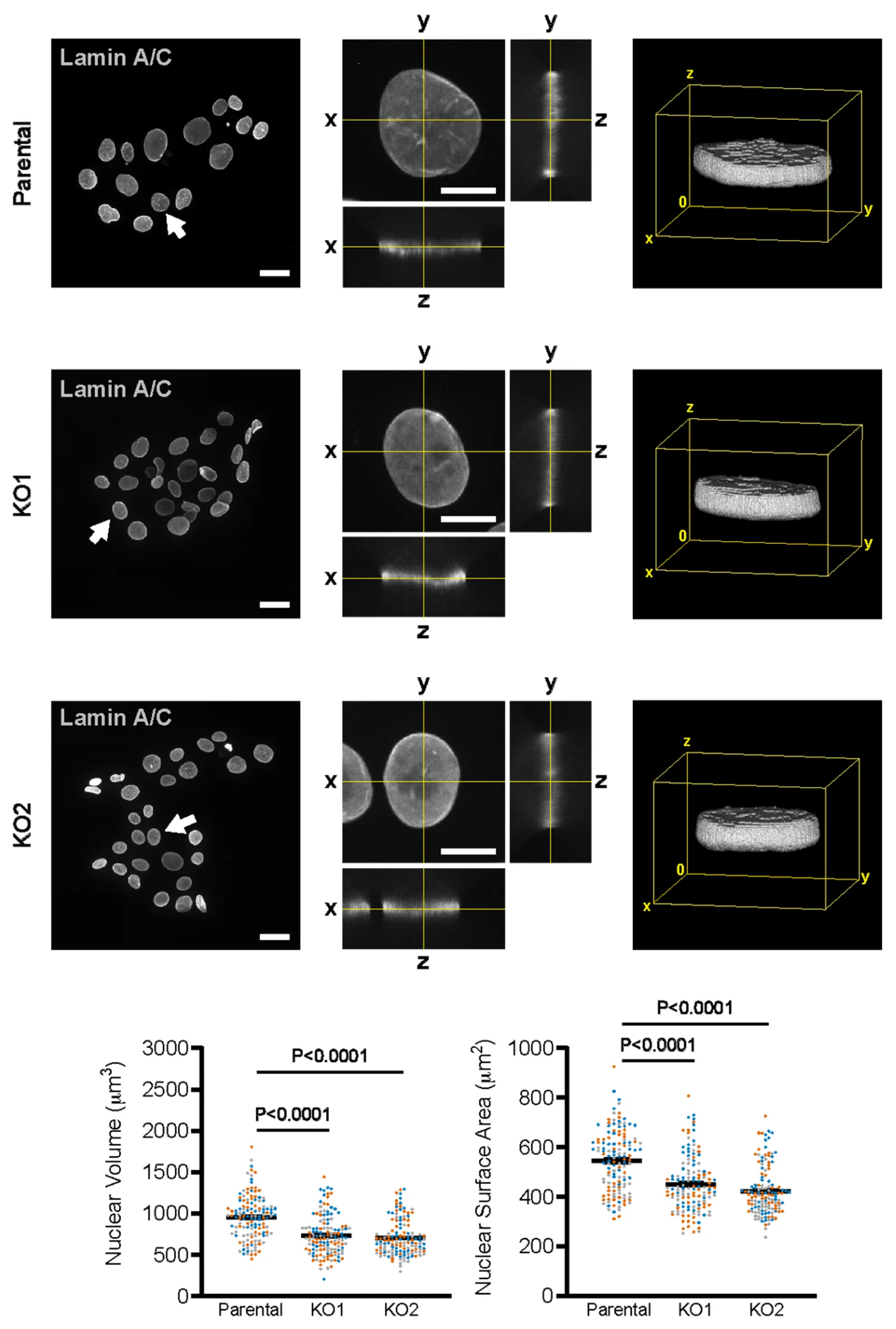
Reduced nuclear dimensions in SCC13 KO cells. Confocal micrographs of nuclei from the indicated cell lines processed with antibodies against lamin A/C, to visualize the nuclear lamina. Arrows identify the nuclei shown at right at higher magnification, in orthogonal views and 3D reconstructions. Scale bar, 10 μm. The graphs show nuclear volume and total nuclear surface area (mean ± SEM; mixed-effects model with Tukey’s post hoc test, n = 3, 45 nuclei scored per experiment). Grey, blue and orange data points are values from a given individual biological replicate.

The morphological characteristics of nuclei influence a variety of cellular processes, including gene expression, migration and cell cycle progression. Nuclear morphology varies among different cell types and is altered in the context of pathologies, such as laminopathies and cancer [34]. Factors that influence nuclear shape comprise the nuclear lamina itself and chromatin modifications, as well as cytoplasmic factors, such as mechanotransduction cascades and the supranuclear F-actin cap, among others [34]. Extracellular substrate stiffness also influences nuclear morphology [35]. Thus, we next determined if *FERMT1* inactivation alters nuclear volume or total surface area, assessed through the analysis of nuclear lamin A/C immunoreactivity confocal stacks. These experiments were conducted using cells seeded on stiff tissue culture polymer surfaces (Young modulus ~ 1 GPa). Nuclear volume in parental cells (953 ± 22.7 μm^3^) was significantly greater than in KO1 and KO2 cells (733 ± 20.6 μm^3^ and 703 ± 18.5 μm^3^, respectively; Figure 6 and Supplemental Table S4). Similarly, total nuclear surface area in parental cells (546.6 ± 10.6 μm^2^) was greater than in KO1 and KO2 cells (450 ± 9.7 μm^2^ and 423 ± 8.5 μm^2^, respectively, Figure 6 and Supplemental Table S4). 

**Figure 7:**
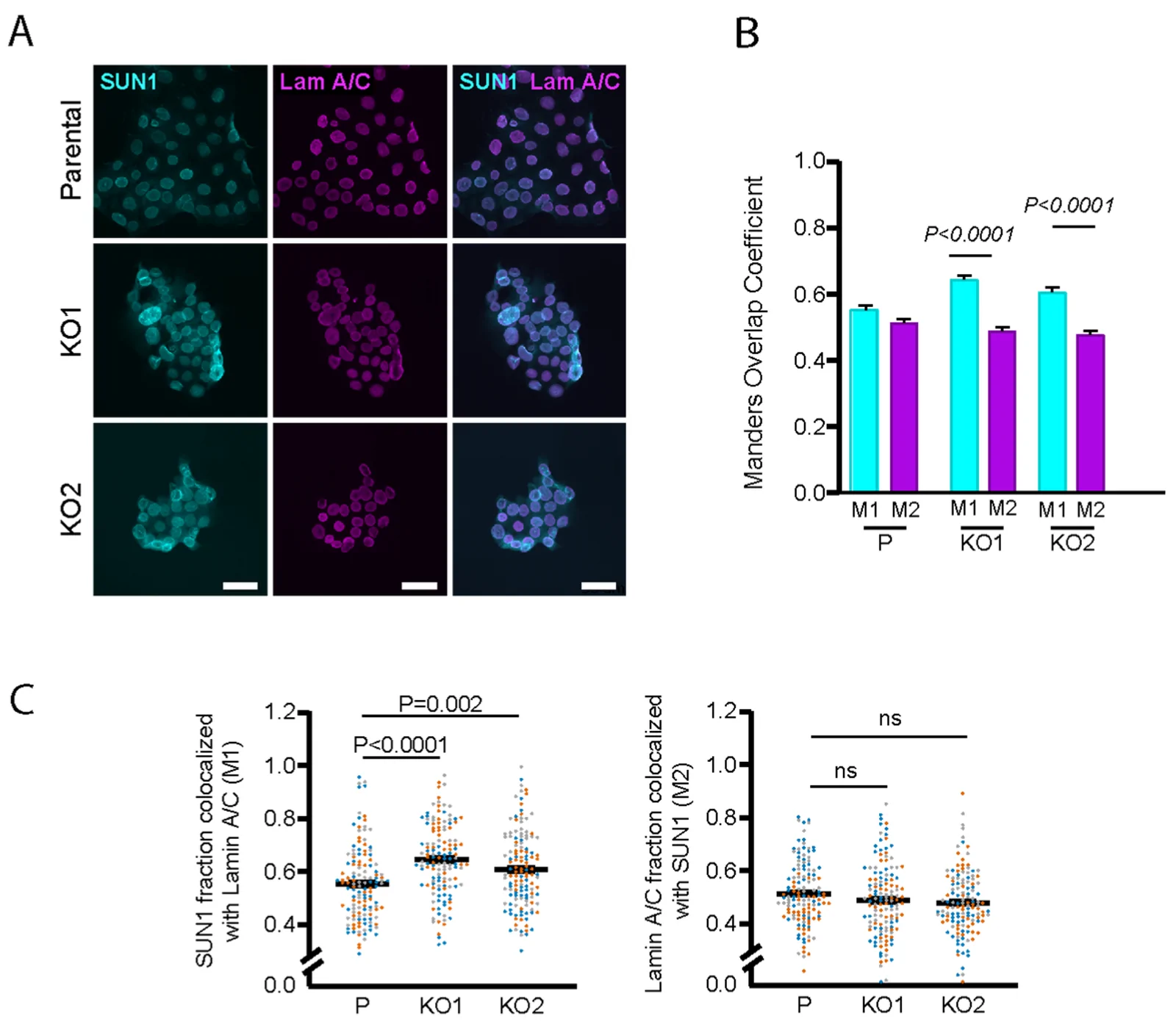
Patterns of lamin A/C and SUN1 nuclear colocalization in Parental and KO SCC13 cells. Cultures of the indicated cell lines were processed for confocal microscopy, and patterns of colocalization were analyzed using Manders’ coefficients. (**A**) Confocal micrographs of nuclei from the indicated cell lines, processed with antibodies against lamin A/C or SUN1. Scale bar, 50 μm. (**B**) Manders’ overlap coefficients in each cell line, indicating the fraction of SUN1 immunoreactivity that colocalizes with lamin A/C (M1) and the fraction of lamin A/C immunoreactivity that colocalizes with SUN1 (M2). (**C**) M1 and M2 coefficients in individual nuclei analyzed. The data in all graphs are expressed as mean ± SEM (mixed-effects model with Tukey’s post hoc test, n = 3, 45 nuclei scored per experiment); ns, not significant. Grey, blue and orange data points are values from a given individual biological replicate.

In epidermal keratinocytes, the nucleus can receive and respond to integrin-dependent mechanical signals from focal adhesions connected to the F-actin cytoskeleton via the LINC protein complex [36,37]. The main components of the LINC complex are KASH-domain proteins, such as nesprins, and SUN proteins, which are anchored in the outer and the inner nuclear membrane, respectively, and associate with each other [38]. Nesprins also bind to cytoplasmic F-actin and the microtubule network, whereas SUN proteins are linked with lamins in the nuclear lamina [39]. Given the key roles of the LINC complex in maintenance of nuclear morphology and the transmission of signals from the plasma membrane to the nucleus, we next examined if absence of Kindlin-1 altered SUN1 distribution. In parental and both KO cells, SUN1 immunoreactivity was mainly detected in the nucleus, although KO1 and KO2 cells also showed weak SUN1 cytoplasmic immunoreactivity (Figure 7A). We then investigated the patterns of co-localization of lamin A/C and SUN1, through the analysis of Manders’ overlap coefficients. We found that the fraction of lamin A/C immunoreactivity that overlapped with SUN1 in parental cells (M2 coefficient, 0.51 ± 0.01) was similar in KO1 and KO2 cells (0.49 ± 0.01 and 0.48 ± 0.01, respectively; Figure 7B,C). However, whereas in parental cells the fraction of SUN1 fluorescence which colocalized with lamin A/C (M1 coefficient) was 0.55 ± 0.01, in KO1 and KO2 cells it was significantly greater (0.65 ± 0.01 and 0.65 ± 0.0125, respectively; Figure 7B,C). Collectively, our observations indicate that Kindlin-1 is important to maintain normal nuclear morphology and suggest that the absence of Kindlin-1 may alter the spatial relationship between SUN1 and lamin A/C in SCC13 nuclei. 

### 3.4. An Intact Integrin-Binding Domain in Kindlin-1 Is Required to Maintain Nuclear Dimensions

**Figure 8:**
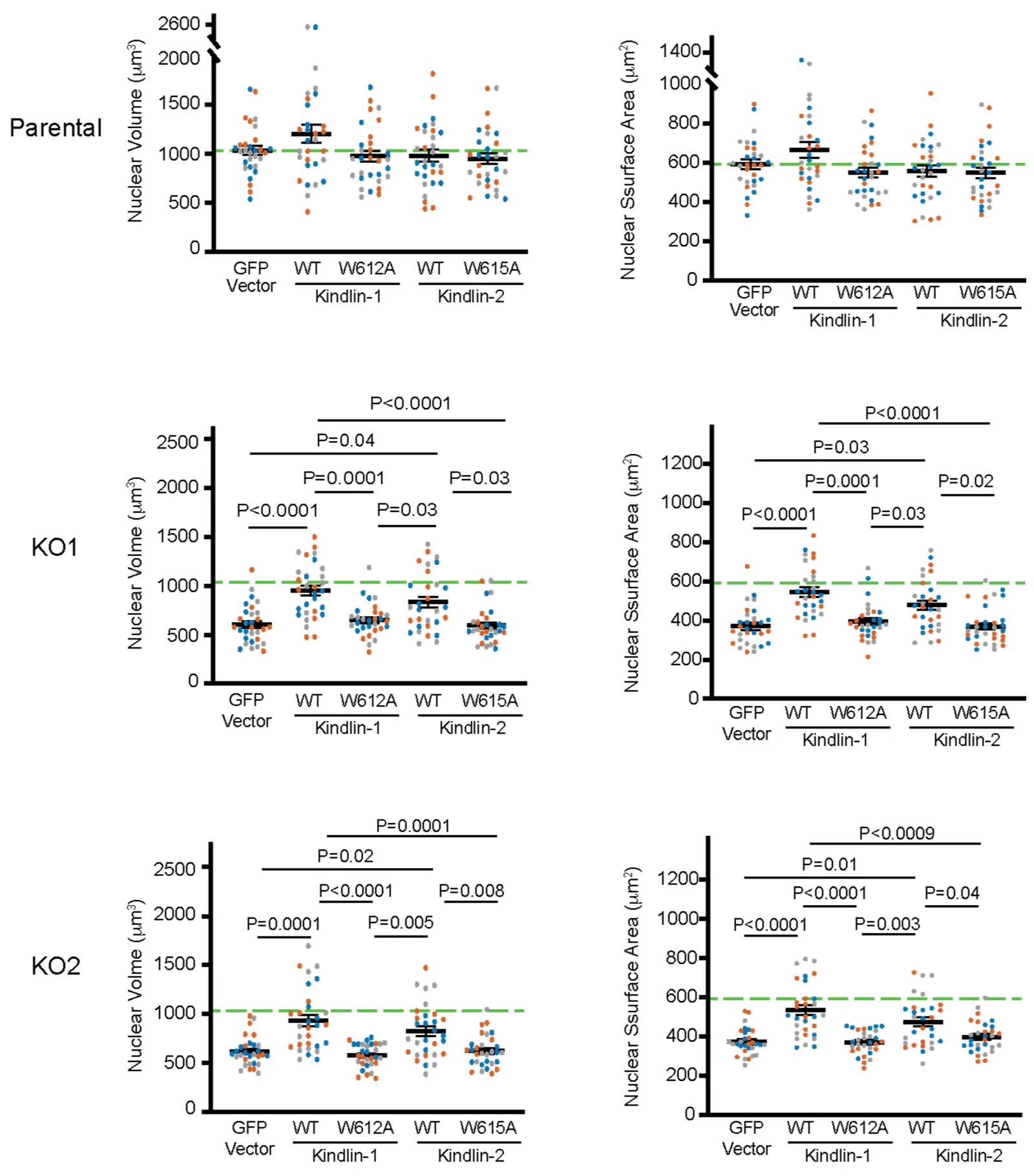
Effect of exogenously expressed Kindlin-1 and Kindlin-2 proteins on nuclear volume and surface area in SCC13 cells. Nuclear volume and total surface area were measured in parental, KO1 and KO2 cells transfected with vectors encoding the indicated proteins. Dashed lines indicate mean volume or area values of parental cells transfected with control GFP vector. Bars represent the mean ± SEM (mixed-effects model with Tukey’s post hoc test, n = 3, 10 nuclei scored per experiment). Grey, blue and orange data points are values from a given individual biological replicate.

Given the link between focal adhesions, F-actin and nuclear morphology, we next investigated if the interaction of Kindlin-1 with integrins at focal adhesions is necessary to maintain nuclear dimensions. Exogenous expression of WT Kindlin-1 in parental cells did not significantly affect nuclear volume or surface area, whereas it restored them in KO1 and KO2 cells to levels comparable to those measured in control GFP vector-expressing parental cells. In contrast, the morphological abnormalities of KO1 and KO2 nuclei expressing Kindlin-1 W612A were maintained, while this mutant protein was without effect in parental SCC13 cells (Figure 8, Supplemental Figures S6–S8, Supplemental Table S5).

To complement these experiments, we also explored whether Kindlin-2 could restore nuclear morphology in KO cells. We found that WT Kindlin-2 only partially restored nuclear dimensions in KO1 and KO2 cells, without inducing any measurable alterations in parental nuclei. Significantly, expression of Kindlin-2 W615A in either parental, KO1 or KO2 cells did not alter nuclear volume or surface area when compared to the corresponding control vector-transfected cells (Figure 8, Supplemental Figures S6–S8, Supplemental Table S5). Thus, Kindlin-1 appears to be an essential component of a mechanoregulatory pathway that links integrins and focal adhesions to the maintenance of nuclear dimensions in SCC13 cells, and this role cannot be completely fulfilled by Kindlin-2. 

### 3.5. Involvement of Active RhoA in Maintenance of Nuclear Morphology

Members of the Rho family of GTPases participate in the regulation of mechanosensing, cytoskeletal remodelling and nuclear morphology [40,41]. To investigate whether they may be involved in the maintenance of nuclear dimensions downstream from Kindlin-1, we exogenously expressed GFP-tagged constitutively active mutant forms of RhoA (G14V), Cdc42 (G12V) and Rac1 (G12V). 

**Figure 9:**
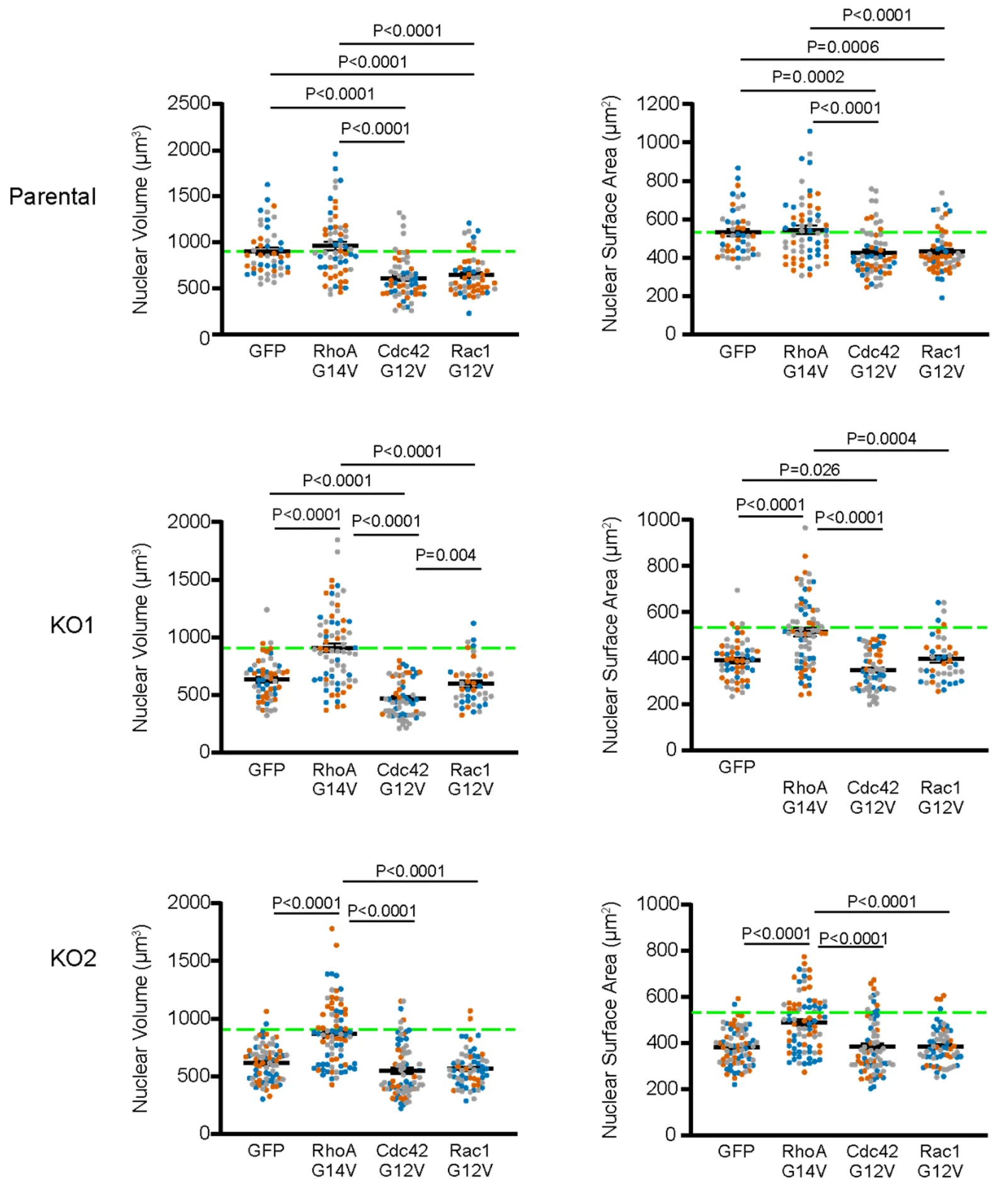
Effect of constitutively active Rho GTPases on nuclear volume and surface area in SCC13 cells. Nuclear volume and total surface area were measured in parental, KO1 and KO2 cells transfected with vectors encoding the indicated GFP-tagged Rho GTPases. Dashed lines indicate mean volume or area values of parental cells transfected with control GFP vector. Bars represent the mean ± SEM (mixed-effects model with Tukey’s post hoc test, n = 3, 11–32 nuclei scored per experiment). Grey, blue and orange data points are values from a given individual biological replicate.

The nuclear volume and surface area of RhoA G14V-expressing parental cells were not significantly different from those in vector-transfected cells. In contrast, nuclear area and volume in KO1 and KO2 cells expressing RhoA G14V were increased to levels comparable to those in parental cells transfected with vectors encoding either GFP or RhoA G14V. Notably, expression of Cdc42 G12V or Rac1 G12V resulted in significant alterations in nuclear morphology, including decreased nuclear volume and surface area, as well as formation of irregularly shaped and/or lobulated nuclei, irrespective of the presence or absence of Kindlin-1 (Figure 9, Supplemental Figure S9, Supplemental Table S6).

The alterations observed in the nuclei of cells expressing constitutively active Rac1 and Cdc42 led us to additionally estimate nuclear circularity values. Parental cells transfected with either control GFP or RhoA-G14V-encoding vectors exhibited similar nuclear circularity values, which were narrowly distributed around the median, with first through third quartile values ranging between 0.80 and 0.85 (Supplemental Figure S10, Supplemental Table S7). Nuclear circularity in KO1 and KO2 cells transfected with control GFP vector was significantly different from values in the corresponding parental cells, with first and third interquartile ranges between 0.74 and 0.84. RhoA-G14V expression in both KO lines yielded similar nuclear circularity values to those in vector-transfected parental cells, with first through third quartile ranges between 0.81 and 0.86. Parental cells expressing Cdc42-G12V or Rac1-G12V displayed significantly lower and more broadly distributed circularity values relative to those in cells transfected with vectors encoding GFP or RhoA-G14V (first through third quartile ranges of 0.63–0.78 and 0.66–0.82, respectively). Similar alterations were observed in KO1 and KO2 cells expressing Cdc42-G12V or Rac1-G12V (Supplemental Figure S10, Supplemental Table S7). These observations are consistent with the notion that RhoA-G14V expression in KO cells can bypass the requirement for Kindlin-1 in the maintenance of normal nuclear morphology, and that the nuclear disruptions induced by constitutively active Cdc42 and Rac1 are independent of Kindlin-1.

**Figure 10:**
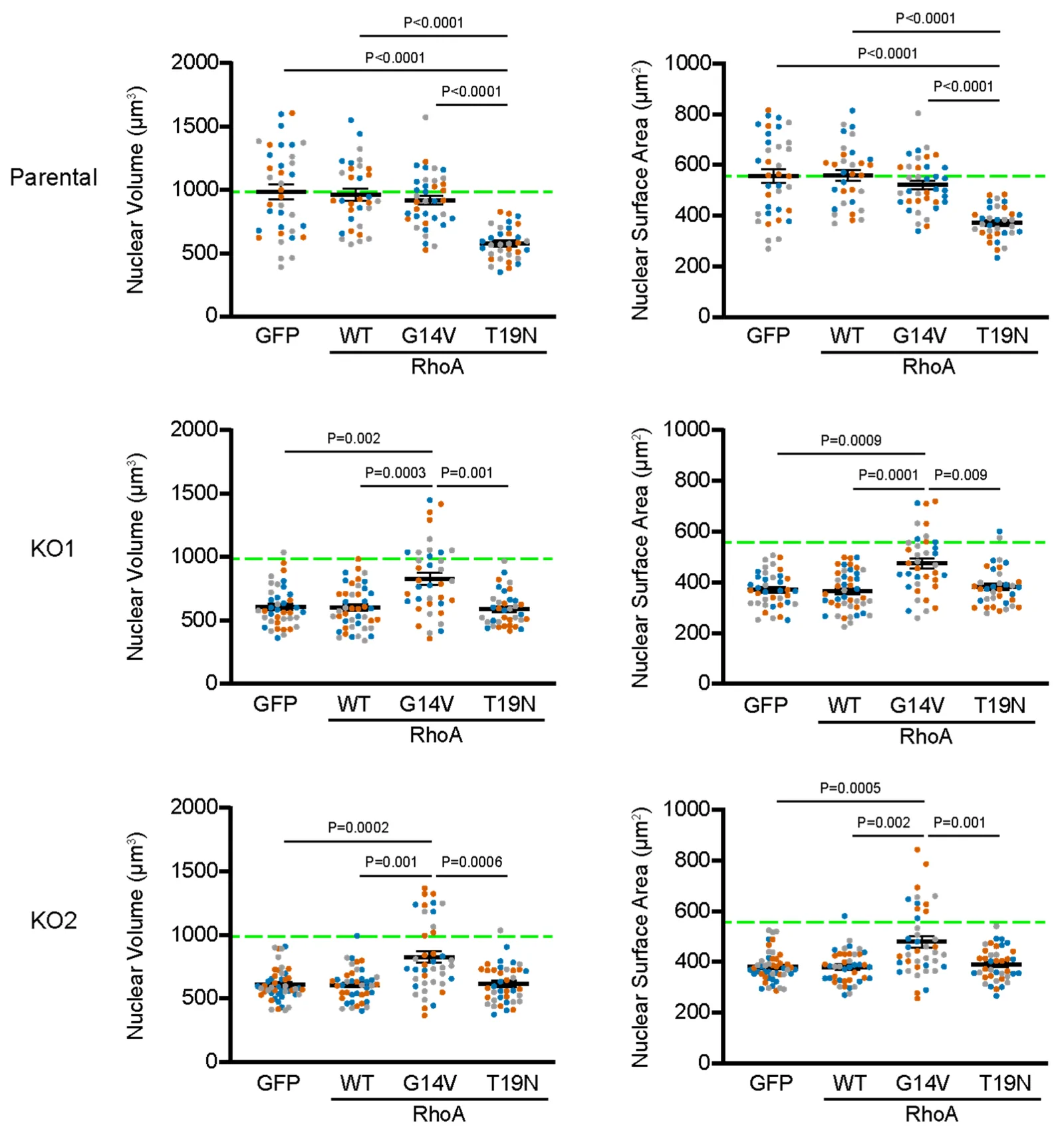
Effect of RhoA GTPases on nuclear volume and surface area in SCC13 cells. Nuclear volume and total surface area were measured in parental, KO1 and KO2 cells transfected with vectors encoding the indicated GFP-tagged RhoA GTPases. Dashed lines indicate mean volume or area values of parental cells transfected with control GFP vector. Bars represent the mean ± SEM (mixed-effects model with Tukey’s post hoc test, n = 3, 10–17 nuclei scored per experiment). WT, wild type. Grey, blue and orange data points are values from a given individual biological replicate.

We next asked whether the observed restoration of nuclear dimensions in KO cells by RhoA-G14V was associated with RhoA activation or overexpression. To this end, we transfected the cells with vectors encoding GFP-tagged wild-type (WT), constitutively active (G14V) or dominant-negative (T19N) RhoA proteins. In parental cells, neither expression of WT nor G14V RhoA altered nuclear volume and surface area compared to control GFP-transfected cells. Remarkably, expression of RhoA-T19N significantly reduced nuclear volume and surface area to values comparable to those observed in GFP-expressing KO cells. In KO1 and KO2 cells, no significant effect on nuclear volume or surface area were observed with exogenous expression of WT or T19N RhoA. However, as previously observed, expression of RhoA-G14V restored nuclear volume and surface area to levels comparable to those measured in GFP-expressing parental cells (Figure 10, Supplemental Figure S11, Supplemental Table S8). Thus, both Kindlin-1 and RhoA modulate nuclear dimensions in SCC13 cells, and the presence of constitutively active RhoA G14V can bypass the nuclear morphology perturbations caused by Kindlin-1 deficiency.

## 4. Discussion

Cells constantly sense and respond to mechanical cues from the extracellular environment and from intracellular tension, through a variety of mechanisms. A major mechanotransduction axis consists of interactions of integrins with extracellular matrix substrates, formation of focal adhesions, modulation of the actin cytoskeleton and activation of downstream cellular responses [10]. Another key element for sensing and responding to mechanical forces is the nucleus [42]. Although nuclear responses to mechanical signals may vary depending on the cell type and its microenvironment, changes in nuclear morphology associated with mechanical tension contribute to the modulation of gene expression, as well as cellular behaviour and phenotype [42]. In this report, we show that Kindlin-1 plays a hitherto unappreciated central role in modulating nuclear morphology. Significantly, this function is dependent on the ability of Kindlin-1 to interact with integrin cytoplasmic domains. Such interactions are also involved in focal adhesion formation and/or maintenance in SCC13 cells.

Kindlins are important components of integrin adhesomes, and epidermal cells express both Kindlin-1 and Kindlin-2. The relative contribution of Kindlin-1 to focal adhesions in cells of epidermal origin appears to depend on the cell model examined. For example, the size and abundance of focal adhesions in primary normal human keratinocytes were reportedly similar to those in primary or immortalized epidermal cells isolated from humans with Kindler syndrome, which lack Kindlin-1 due to inactivating *FERMT1* mutations [5,43]. Analogous observations were reported in studies comparing Kindlin-1-expressing and -deficient HaCaT cells [44]. In contrast, decreased focal adhesion abundance was reported in a different study using another line of immortalized Kindlin-1-deficient human keratinocytes [45]. Our findings indicate that Kindlin-1 plays an essential role in normal focal adhesion assembly and/or maintenance in SCC13 epidermal squamous carcinoma cells, which cannot be completely fulfilled by Kindlin-2.

The link between modulation of F-actin cortical and stress fibres, focal adhesions and kindlins is well established [2,22]. Less well characterized are the mechanisms that impact the supranuclear F-actin cap. Nevertheless, vimentin and focal adhesion kinase (FAK) have been identified as important regulatory components of those distinct focal adhesions that participate in actin cap assembly [30], as well as Rac1 and its downstream effectors STEF and TIAM2 [46]. Our studies now reveal that the absence of Kindlin-1 causes not only significant alterations in F-actin bundles associated with stress fibres, but also in those localized to supranuclear cytoplasmic regions, which likely include the perinuclear actin cap and any cortical actin fibres that may be present in these cells. Kindlins are adaptor proteins that regulate various signalling pathways that modulate cytoskeletal dynamics, including activation of Src kinase and Rho GTPases downstream from integrin clustering [2]. The exact role of Kindlin-1 in modulating the supranuclear actin cap remains to be defined, as our experiments did not address whether its formation or its stabilization is impaired in the absence of Kindlin-1.

It is now recognized that physical connections between perinuclear actin cap filaments and the nuclear envelope are key determinants of nuclear architecture and morphology [31]. Appropriate regulation of nuclear shape is critical for cell motility and polarization, and its disruption is linked to human pathologies including cancer and various genetic diseases [47,48]. Nevertheless, the factors and mechanisms that govern nuclear morphology remain poorly understood. We have uncovered a hitherto unappreciated critical role for Kindlin-1 in the regulation of nuclear volume and total surface area. Significantly, this property of Kindlin-1 requires its binding to β integrin cytoplasmic domains, and cannot be executed, at least fully, by Kindlin-2. The alterations in focal adhesions in Kindlin-1 KO cells may lead to impaired attachment to the extracellular matrix, together with an altered supranuclear actin cytoskeleton. These events, in turn, might contribute to nuclear morphological changes. Consistent with this possibility, cell detachment from extracellular substrates leads to marked decreases in nuclear volume in a variety of cell types [49]. β1 and β3 integrins bind and are activated by Kindlin-1 [20]. Epidermal cells express α2β1 and α3β1 integrins, which mediate attachment to collagen and laminin 332, respectively [50]. In our studies, SCC13 cells were not seeded on ECM substrate-coated surfaces. However, SCC13 cells and epidermal keratinocytes secrete laminin 332, suggesting that Kindlin-1 may be an important component in a pathway that involves modulation of nuclear characteristics through β1 integrin attachment to and activation by laminin 332 [51]. 

In addition to mediating adhesion to extracellular substrates, integrins are central components of the mechanosensing and mechanotransduction machinery [10]. Cells are constantly subjected to mechanical forces from the environment and from intrinsically generated tension generated by the cytoskeleton. Interactions between integrins and the actin cap act as sensors and modulators of force transmission from the cell membrane to the nuclear envelope, via LINC complexes [52,53]. Changes in nuclear morphology and LINC proteins modulate nuclear pore complex function, translocation of mechanosensitive transcription factors, chromatin architecture and gene expression [53]. Although our study did not address the functional consequences of the observed nuclear volume changes in the absence of Kindlin-1, the alterations we observed in the co-localization patterns of Lamin A/C and SUN1 might reflect LINC complex perturbations., which will be an important area for future research. 

Mechanical tension exerted on the cell surface by stiff substrates or other stimuli are sensed by integrins, promoting their engagement, clustering and activation [1]. Activated integrins associate with kindlins and other adaptor proteins. In addition, downstream from integrins, and irrespective of the nature of the mechanical stimulus, RhoA signalling modulates the actin cytoskeleton and the nucleus [54]. In epidermal keratinocytes, RhoA activation during formation of intercellular adhesions requires Kindlin-1 [43]. Although in the present study we have not addressed whether integrin-mediated activation of RhoA is impaired in Kindlin-1-deficient KO cells, our data reveal that nuclear volume and total surface area in Kindlin-1 KO cells can be restored by exogenously expressed constitutively active, but not wild-type, RhoA. In addition, interfering with RhoA-modulated pathways by expressing dominant-negative RhoA T17N did not further alter nuclear dimensions in Kindlin-1-deficient KO cells, although it decreased nuclear volume and total surface area in a Kindlin-1-expressing cell background. Together, these observations indicate that both Kindlin-1 and RhoA are important to maintain nuclear morphology. The possibility that RhoA signals that contribute to the nuclear phenotype might function directly downstream from Kindlin-1 will be an important area for future investigation.

In summary, our work is the first to uncover, to our knowledge, a key essential role for Kindlin-1 in the modulation of nuclear morphology in SCC13 epidermal squamous carcinoma cells. A limitation of this work is the use of a single cutaneous SCC cell line. Thus, an important area for future research will be to determine whether Kindlin-1 plays different roles in the preservation of nuclear morphology in distinct subsets of epidermal squamous cell carcinomas. Given the importance of the reciprocal regulation of nuclear function and cell behaviour, as well as nuclear size and plasticity for tumour cell motility and metastasis, our findings are likely to have significant implications for tumourigenesis.

## 5. Conclusions

Our study identifies Kindlin-1 as a key modulator of nuclear dimensions and morphology downstream from the integrin adhesome in transformed epidermal SCC13 cells. We also demonstrate the ability of a constitutively active RhoA mutant protein to restore these nuclear characteristics in the absence of Kindlin-1. These observations warrant further studies to determine whether RhoA activation in response to mechanical signals requires Kindlin-1, and whether Kindlin-1 fulfils these functions in subsets of epidermal squamous carcinoma cells. A better understanding of these processes might also provide insight into a potential susceptibility of cutaneous squamous cell carcinomas to therapeutic interventions targeting this pathway.

## Data Availability

All data supporting the reported results are included in the manuscript.

## Supplementary Materials

The following supporting information can be downloaded at: https://www.mdpi.com/article/10.3390/cells15211991/s1, Supplemental Table S1: Antibodies and fluorescence reagents used in these studies; Supplemental Table S2: Effect of exogenous expression of Kindlin-1 proteins on focal adhesions; Supplemental Table S3: Effect of exogenous expression of Kindlin-2 proteins on focal adhesions; Supplemental Table S4: Nuclear dimensions in parental and Kindlin-1 KO SCC13 cells; Supplemental Table S5: Effect of exogenous expression of Kindlin proteins on nuclear volume and surface area; Supplemental Table S6: Effect of exogenous expression of constitutively active Rho GTPases on nuclear volume and total surface area; Supplemental Table S7: Effect of exogenous expression of constitutively active Rho GTPases on nuclear circularity; Supplemental Table S8: Effect of exogenous expression of RhoA GTPases on nuclear volume and total surface area. Figure S1: Effect of *FERMT1* inactivation on focal adhesions; Figure S2: Effect of Kindlin-1 proteins on focal adhesions in SCC13 KO2 cells; Figure S3: Expression of FLAG-tagged Kindlin proteins; Figure S4: Effect of Kindlin-2 proteins on focal adhesions in SCC13 KO2 cells; Figure S5: Alterations in microtubule networks in SCC13 KO cells; Figure S6: Effect of exogenous Kindlin proteins on nuclear dimensions in parental SCC13 cells; Figure S7: Effect of exogenous Kindlin proteins on nuclear dimensions in SCC13 KO1 cells; Figure S8: Effect of exogenous Kindlin proteins on nuclear dimensions on SCC13 KO2 cells; Figure S9: Effect of constitutively active GTPases on nuclear dimensions in parental, KO1 and KO2 cells; Figure S10: Effect of constitutively active GTPases on nuclear morphology in SCC13 cells; Figure S11: Effect of RhoA GTPase proteins on nuclear dimensions; Supplemental Methods: Measurement of 2-dimensional cell area from confocal z-stack image projections.

## Abbreviations

The following abbreviations are used in this manuscript:

cSCC	Cutaneous squamous cell carcinoma
ECM	Extracellular matrix
FAK	Focal adhesion kinase
GPa	Gigapascal
LINC	Linker of Nucleoskeleton and Cytoskeleton
SEM	Standard error of the mean
WT	Wild type
