# Supplementary material for: Kindlin-1 Links Integrins and Maintenance of Nuclear Shape in Cutaneous Squamous Carcinoma Cells
Source: Cells. 2026 Oct 31;15(21):1991. doi: 10.3390/cells15211991 (PMC13761988; doi:10.3390/cells15211991)

Supplemental Table S1. Antibodies and fluorescence reagents used in these studies[1].

Antibody	Dilution	Source
β-Tubulin (β-5 chain)	IB 1:3000<br>IF 1:100	Developmental Studies Hybridoma Bank, University of Iowa, Iowa City, IO, USA (E7)
FLAG (DYKDDDDK)	IF 1:800	Cell Signaling Technology, Danvers, MA, USA (8146T)
FLAG (DYKDDDDK)	IB: 1:1000<br>IF 1:800	Cell Signaling Technology (14793S)
Lamin A/C	IF 1:200	Cell Signaling Technology (4777S)
SUN1	IF 1:250	Novus Biologicals, Centennial, CO, USA(NBP1-87396)
Phospho-paxillin (Tyr118)	IF 1:100	Cell Signaling Technology (69363S)
AlexaFluor™ 488-conjugated anti-mouse IgG	IF 1:500	Thermo Fisher Scientific, Waltham, MA, USA (A-11001)
AlexaFluor™ 488-conjugated anti-rabbit IgG	IF 1:500	Thermo Fisher Scientific (A-11008)
AlexaFluor™ 594-conjugated anti-mouse IgG	IF 1:500	Jackson ImmunoResearch, West Grove, PA, USA (115-585-062)
AlexaFluor™ 555-conjugated anti-rabbit IgG	IF 1:250	Thermo Fisher Scientific (A-21428)
HRP-conjugated anti-mouse IgG	IB 1:5000	Jackson ImmunoResearch (115-035-146)
HRP-conjugated anti-rabbit IgG	IB 1:5000	Jackson ImmunoResearch (111-035-144)
AlexaFluor™ 488-conjugated phalloidin	IF 1:800	Cell Signaling Technology (8878S)

[1] IB, immunoblot; IF, immunofluorescence microscopy.

Supplemental Table S2. Effect of exogenous expression of Kindlin-1 proteins on focal adhesions.

**Number of focal adhesions per cell (mean ± SEM)**			
**Construct**	**Parental**	**KO1**	**KO2**
Vector	46.6 ± 5.6	10.5 ± 1.6[1]	6.9 ± 1.0[1]
WT	65.3 ± 8.0	35.7 ± 4.9[2]	33.2 ± 4.9[2]
W612A	38.0 ± 5.0	11.1 ± 1.6[1]	8.9 ± 1.5[1]
**Focal adhesion surface area (μm²; mean ± SEM)**			
Vector	0.514 ± 0.021	0.439 ± 0.022[3]	0.413 ± 0.019[3]
WT	0.691 ± 0.03[3]	0.570 ± 0.023[4]	0.528 ± 0.021[4]
W612A	0.570 ± 0.022	0.429 ± 0.017[3]	0.392 ± 0.018[3]

[1] P<0.0001 relative to parental cells transfected with vector.

[2] P<0.0001 relative to corresponding KO cells transfected with vector.

[3] P<0.01 relative to parental cells transfected with vector.

[4] P<0.003 relative to corresponding KO cells transfected with vector.

Supplemental Table S3. Effect of exogenous expression of Kindlin-2 proteins on focal adhesions.

Number of focal adhesions per cell (mean ± SEM)			
**Construct**	**Parental**	**KO1**	**KO2**
Vector	40.0 ± 3.6	12.4 ± 1.5[1]	11.7 ± 1.9[1]
WT	44.9 ± 4.7	24.5 ± 3.5[2]	24.9 ± 3.6[2]
W615A	22.6 ± 3.3[1]	10.1 ± 1.4[1]	7.2 ± 0.9[1]
**Focal adhesion surface area (μm²; mean ± SEM)**			
Vector	0.557 ± 0.019	0.410 ± 0.014[3]	0.389 ± 0.019[3]
WT	0.546 ± 0.019	0.491 ± 0.015 [3,4]	0.436 ± 0.014[3]
W615A	0.443 ± 0.015[3]	0.383 ± 0.011[3]	0.373 ± 0.012[3]

[1] P<0.02 relative to parental cells transfected with vector.

[2] P<0.005 relative to corresponding KO cells transfected with vector.

[3] P<0.0001 relative to parental cells transfected with vector.

[4] P<0.0009 relative to corresponding KO cells transfected with vector.

Supplemental Table S4. Nuclear dimensions in parental and Kindlin-1 KO SCC13 cells.

Cell Line	Nuclear Volume (μm³; mean ± SEM)	Nuclear Surface Area (μm²; mean ± SEM)
Parental	953.1 ± 22.7	545.6 ± 10.6
KO1	733.4 ± 20.6[1]	450.3 ± 9.7[1]
KO2	705.1 ±18.5[1]	423.1 ± 8.5[1]

[1] P<0.0001, relative to parental cells

Supplemental Table S5. Effect of exogenous expression of Kindlin proteins on nuclear volume and surface area

Nuclear Volume (μm³; mean ± SEM)			
**Construct**	**Parental**	**KO1**	**KO2**
GFP Vector	1034.3 ± 47.2	606.3 ± 35.9[1]	617.0 ± 27.3[1]
Kindlin -1 WT	1203.6 ± 91.3	950.8 ± 50.8[2]	931.5 ± 58.1[2]
Kindlin-1 W612A	974.80 ± 54.9	626.7 ± 25.3[1]	578.8 ± 22.4[1]
Kindlin-2 WT	978.56 ± 59.8	803.0 ± 48.5[1,2]	807.3 ± 43.3[1,2]
Kindlin-2 W615A	948.39 ± 55.9	594.9 ± 33.6[1]	623.9 ± 29.5[1]
**Total Nuclear Surface Area (μm²; mean ± SEM)**			
GFP Vector	591.6 ± 23.4	370.2 ± 18.0[3]	372.5 ± 12.1[3]
Kindlin -1 WT	664.9 ± 39.0	545.2 ± 23.9[4]	533.1 ± 24.8[4]
Kindlin-1 W612A	548.8 ± 24.8	382.0 ± 14.4[3]	369.6 ± 10.9[3]
Kindlin-2 WT	556.3 ± 28.5	466.6 ± 21.8[3,4]	469.0 ± 20.0[3,4]
Kindlin-2 W615A	547.8 ± 27.4	369.8 ± 16.9[3]	397.5 ± 14.4[3]

[1] P<0.04 relative to parental cells transfected with control vector.

[2] P<0.04 relative to corresponding KO cells transfected with control vector.

[3] P<0.02 relative to parental cells transfected with control vector.

[4] P<0.03 relative to corresponding KO cells transfected with control vector.

Supplemental Table S6. Effect of exogenous expression of constitutively active Rho GTPases on nuclear volume and total surface area

Nuclear Volume (μm³; mean ± SEM)			
**Construct**	**Parental**	**KO1**	**KO2**
GFP	905.1 ± 38.4	632.4 ± 24.5[1]	613.9 ± 18.5[1]
RhoA G14V	961.2 ± 45.0	908.9 ± 39.0[2]	865.8 ± 31.5[2]
Rac1 G12V	612.7 ± 30.7[1]	469.4 ± 21.4[1, 2]	550.3 ± 28.0[1]
Cdc42 G12V	647.2 ± 30.4[1]	596.2 ± 29.8[1]	564.4 ± 19.8[1]
**Total Nuclear Surface Area (μm²; mean ± SEM)**			
GFP	531.9 ± 18.2	391.3 ± 12.0[3]	381.7 ± 9.3[3]
RhoA G14V	545.3 ± 21.1	513.2 ± 18.0[4]	489.7 ± 13.9[4]
Rac1 G12V	425.6 ± 16.3[3]	347.8 ± 11.4[3, 4]	383.1 ± 14.1[3]
Cdc42 G12V	431.9 ± 15.6[3]	396.8 ± 15.7[3]	384.2 ± 10.3[3]

[1] P<0.0001 relative to parental cells transfected with GFP vector.

[2] P<0.0001 relative to corresponding KO cells transfected with GFP vector.

[3]P<0.0006 relative to parental cells transfected with GFP vector.

[4]P<0.03 relative to corresponding KO cells transfected with GFP vector.

Supplemental Table S7. Effect of exogenous expression of constitutively active Rho GTPases on nuclear circularity

Quartile	GFP	RhoA G14V	Cdc42 G12V	Rac1 G12V
**Parental SCC13 cells**				
25th	0.81	0.80	0.63	0.66
50th (Median)	0.82	0.82	0.69	0.72
75th	0.85	0.84	0.78	0.82
Mean ± SD	0.82 ± 0.03	0.81 ± 0.03	0.68 ± 0.10[1]	0.73 ± 0.09[1]
**KO1 cells**				
25th	0.76	0.81	0.56	0.67
50th (Median)	0.80	0.84	0.64	0.76
75th	0.83	0.86	0.69	0.80
Mean ± SD	0.80 ± 0.05	0.83 ± 0.04[1]	0.64 ± 0.10[1]	0.73 ± 0.10[1]
**KO2 cells**				
25th	0.74	0.80	0.54	0.67
50th (Median)	0.80	0.83	0.64	0.73
75th	0.84	0.84	0.72	0.78
Mean ± SD	0.79 ± 0.06	0.82 ± 0.03[1]	0.62 ± 0.12[1]	0.71 ± 0.08[1]

[1] P<0.004 relative to corresponding cell line transfected with control GFP vector.

Supplemental Table S8. Effect of exogenous expression of RhoA GTPases on nuclear volume and total surface area

Nuclear Volume (μm³; mean ± SEM)			
**Construct**	**Parental**	**KO1**	**KO2**
GFP	985.2 ± 57.0	606.9 ± 25.7[1]	608.7 ± 18.3[1]
RhoA WT	963.0 ± 46.0	598.8 ± 25.4[1]	606.2 ± 20.0[1]
RhoA G14V	920.8 ± 35.8	827.1 ± 46.9[2]	826.1 ± 44.3[2]
RhoA T19N	578.9 ± 22.4[*]	589.9 ± 22.8[1]	614.4 ± 23.9[1]
**Total Nuclear Surface Area (μm²; mean ± SEM)**			
GFP	556.7 ± 26.6	370.5 ± 11.1[1]	380.5 ± 8.6[1]
RhoA WT	559.7 ± 21.6	364.3 ± 11.8[1]	379.8 ± 9.8[1]
RhoA G14V	521.1 ± 16.5	474.2 ± 19.8[3]	478.7 ± 21.0[3]
RhoA T19N	371.8 ± 11.1[†]	381.9 ± 13.0[1]	389.4 ± 10.0[1]

[1] P<0.0001 relative to parental cells transfected with GFP vector.

[2] P<0.002 relative to corresponding KO cells transfected with GFP vector.

[3]P<0.0009 relative to corresponding KO cells transfected with GFP vector.

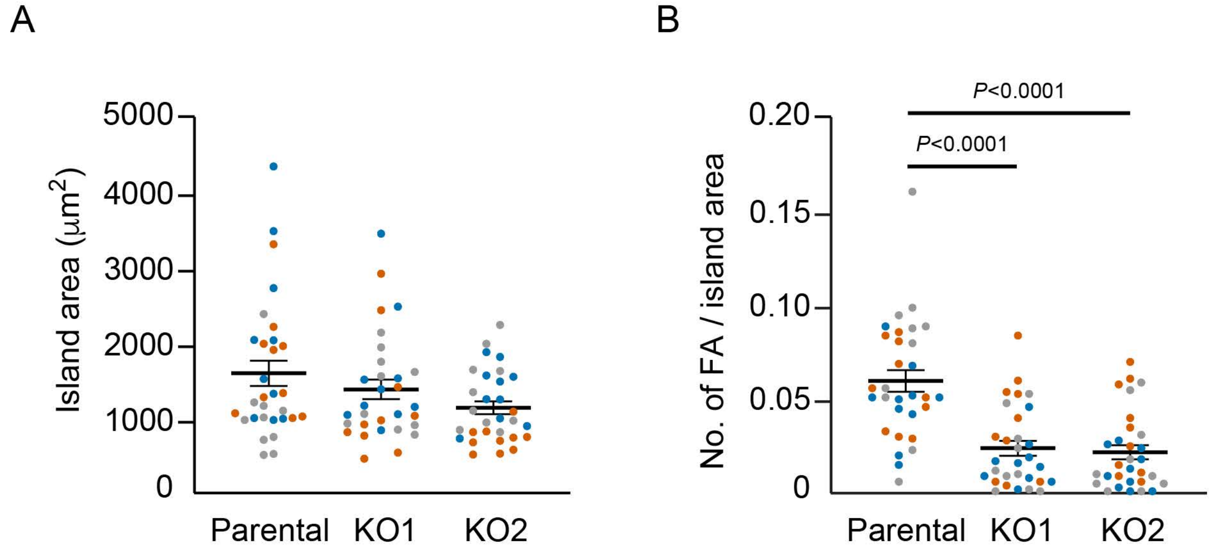

Figure S1. Effect of *FERMT1* inactivation on focal adhesions. (A) The total surface area of islands containing 4 cells in the indicated lines were measured.The graphs show values for individual islands, with the bars representing the mean + SEM. (B) The number of focal adhesions (FA) per 4-cell island were normalized to island surface area. Values shown represent the mean + SEM, and significance was analyzed using a mixed-effects model with Tukey's post-hoc test.(n-3; ten islands scored per experiment). Gray, blue and orange data points in both graphs represent values from a given individual biological replicate.

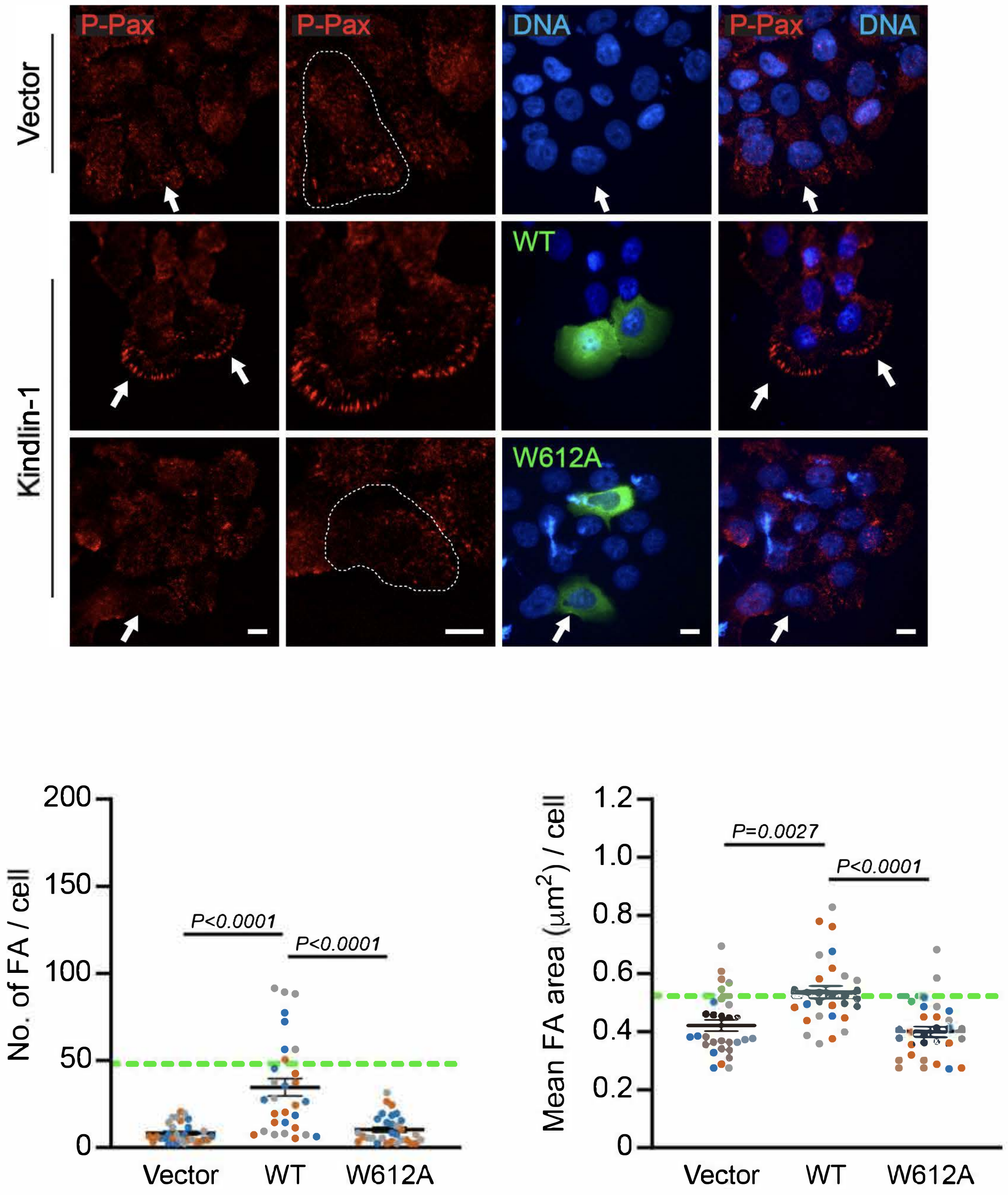

Figure S2. Effect of Kindlin-1 proteins on focal adhesions in SCC13 KO2 cells. Cells were transfected with vectors encoding FLAG-tagged wild type (WT) or W612A Kindlin-1. Twenty-eight hours after transfection, the cells were processed for immunofluorescence microscopy, using anti-phospho-paxillin (P-Pax) antibodies and Hoechst 33342 to visualize, respectively, focal adhesions and nuclear DNA. Arrows and dashed lines indicate transfected cells. Scale bar, 10 μm. The graphs show values for individual cells, with the bars representing mean + SEM. Green dashed lines indicate the mean values of SCC13 Parental cells transfected with empty vector (mixed-effects model with Tukey's post-hoc test, n-3; ten cells scored per experiment). Gray, blue and orange data points represent values from a given individual biological replicate.

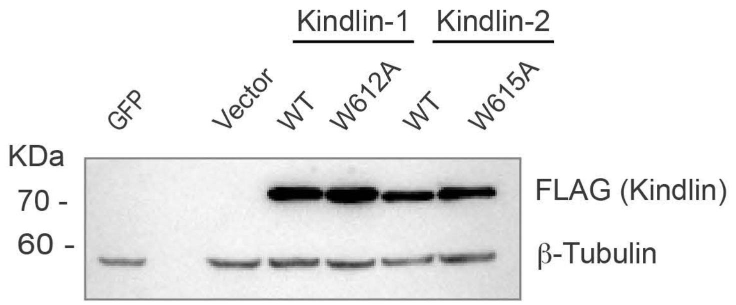

Figure S3.Expression of FLAG-tagged Kindlin proteins. Vectors encoding green fluorescent protein (GFP) or the indicated Kindlin proteins were transfected into IMDF cells. Protein lysates were prepared 36 h after transfection, and analyzed by immunoblot with anti-FLAG antibodies (to detect exogenous Kindlin proteins) or with anti-β-Tubulin antibodies, as loading control.

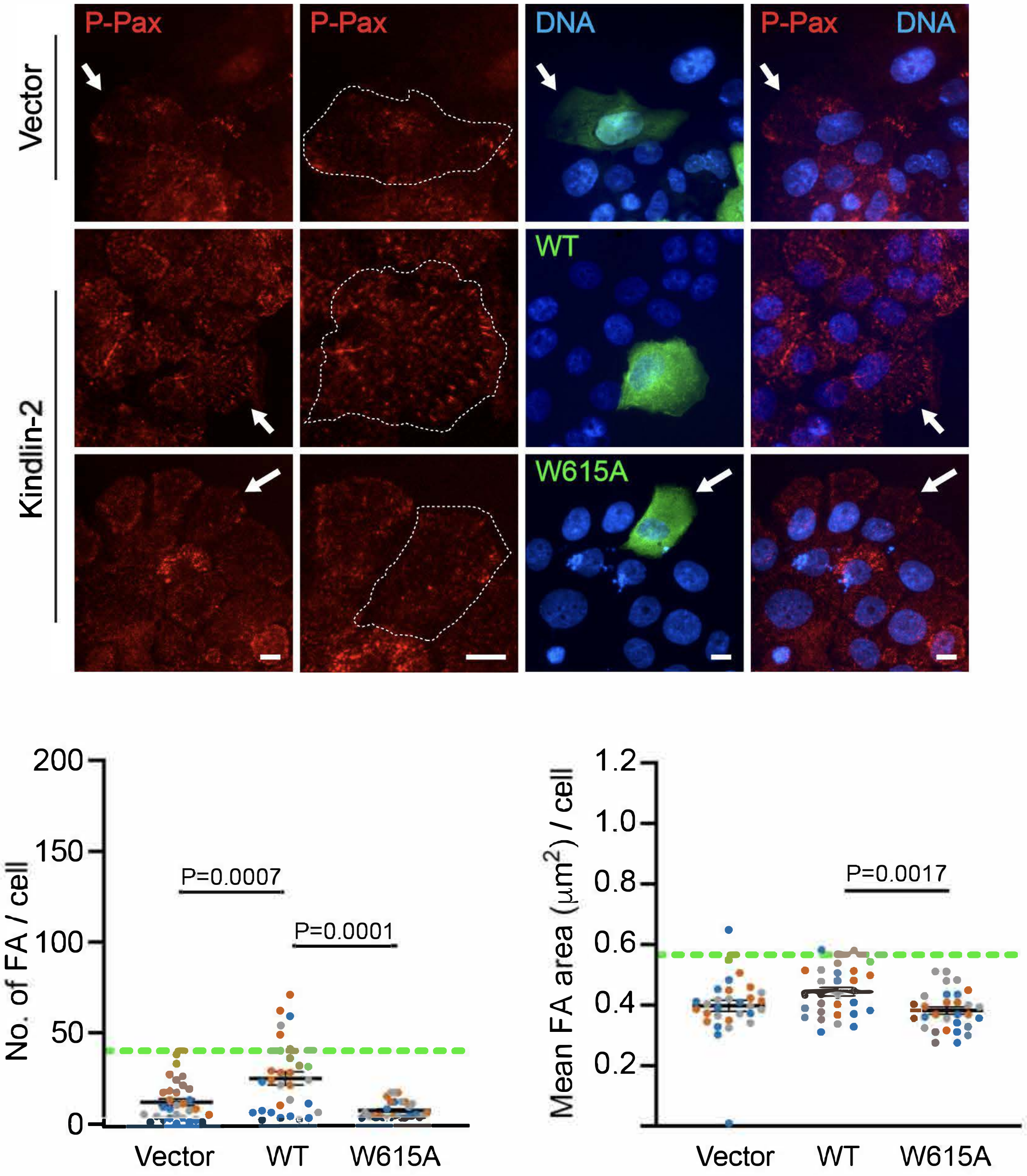

Figure S4. Effect of Kindlin-2 proteins on focal adhesions in SCC13 KO2 cells. Cells were transfected with vectors encoding FLAG-tagged wild type (WT) or W615A Kindlin-2. Twenty-eight hours after transfection, the cells were processed for immunofluorescence microscopy, using anti-phospho-paxillin (P-Pax) antibodies and Hoechst 33342 to visualize, respectively, focal adhesions and nuclear DNA. Arrows and dashed lines indicate transfected cells. Scale bar, 10 μm. The graphs show values for individual cells, with the bars representing mean + SEM. Green dashed lines indicate the mean values of SCC13 Parental cells transfected with empty vector (mixed-effects model with Tukey's post-hoc test, n-3; ten cells scored per experiment). Gray, blue and orange data points represent values from a given individual biological replicate.

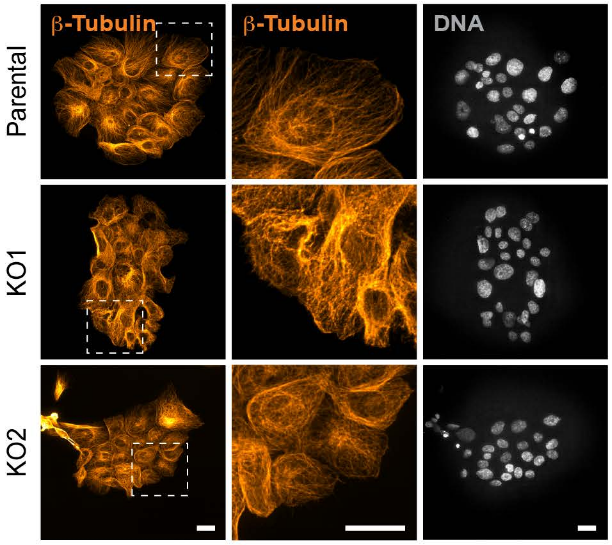

Figure S5. Alterations in microtubule networks in SCC13 KO cells. Confocal micrograph maximum intensity projections of the indicated cell lines, processed with anti-β-tubulin antibodies and Hoechst 33342 to visualize, respectively, microtubules and nuclear DNA. Boxed areas are shown at higher magnification in the micrograph at right. Scale bar, 25 μm.

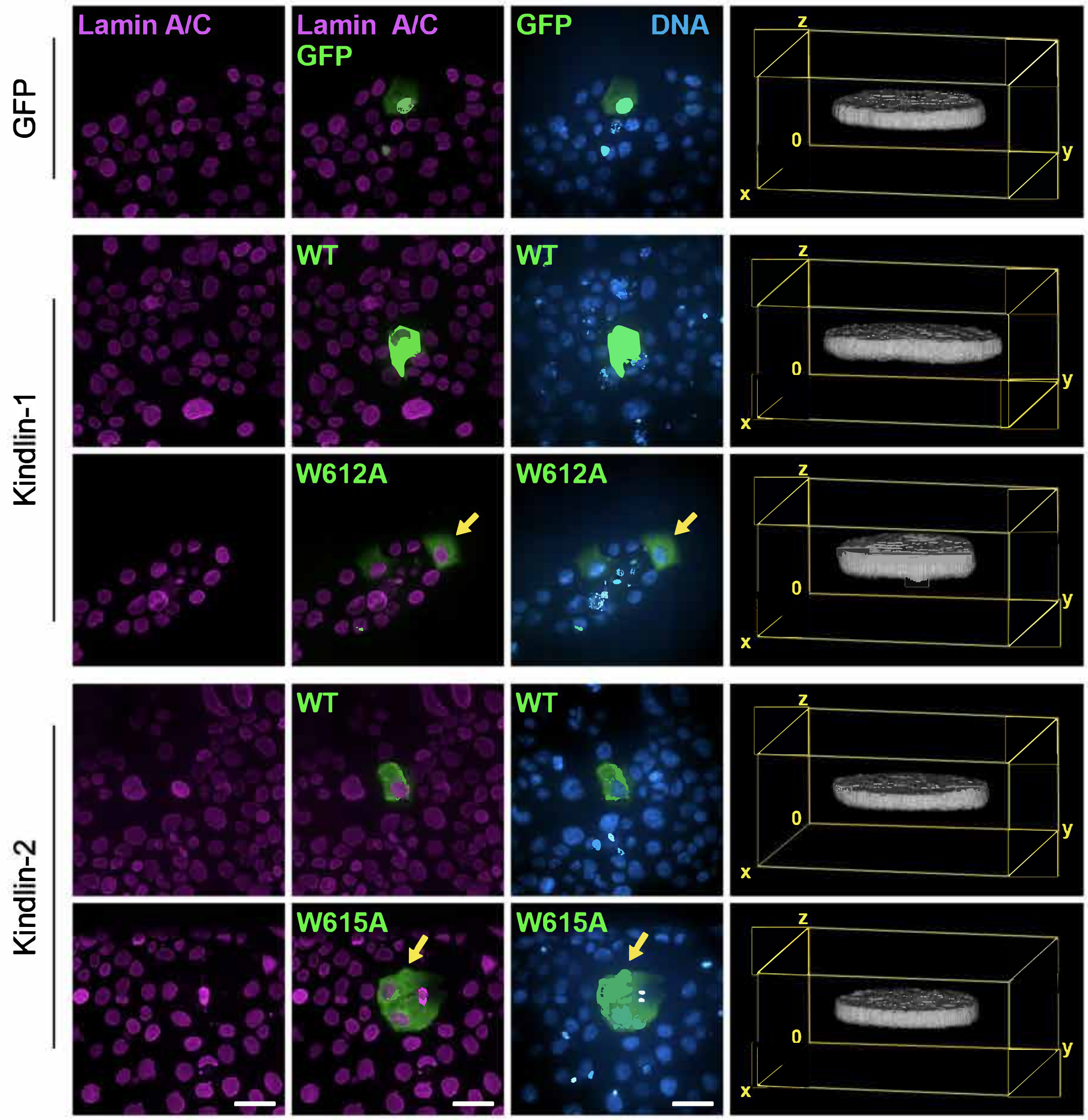

Figure S6. Effect of exogenous Kindin proteins on nuclear dimensions in parental SCC13 cells. Cells were transfected with vectors encoding GFP or the indicated FLAG-tagged Kindlin proteins.Twenty-eight hours after transfection, the cells were processed for confocal microscopy, using anti-lamin A/C antibodies and Hoechst 33342 to visualize, respectively, the nuclear lamina and nuclear DNA. Arrows indicate transfected cells, with their nuclei shown in a 3D reconstruction at right. Scale bar, 50 μm.

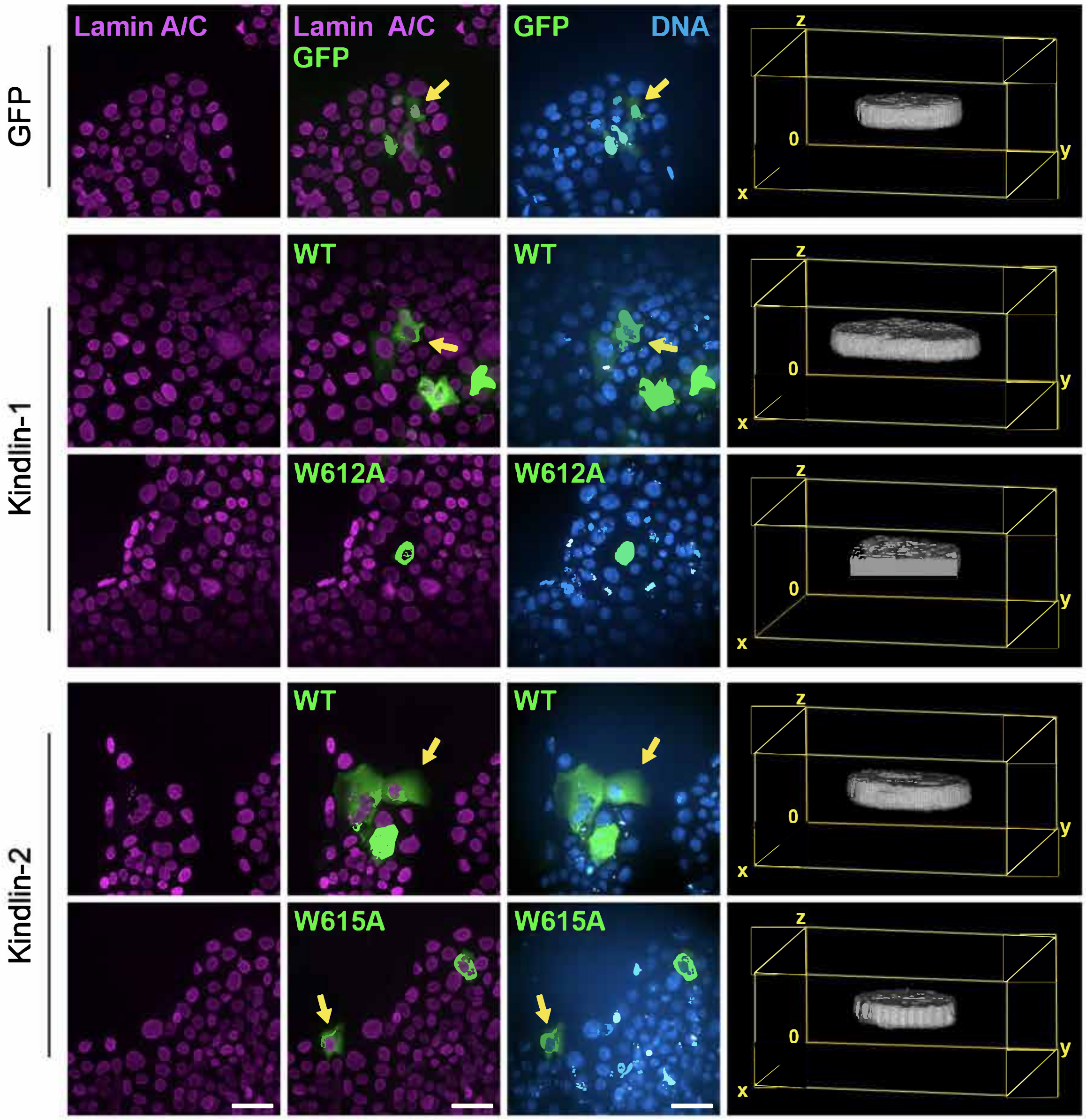

Figure S7. Effect of exogenous Kindlin proteins on nuclear dimensions in SCC13 KO1 cells. Cells were transfected with vectors encoding GFP or the indicated FLAG-tagged Kindlin proteins.Twenty-eight hours after transfection, the cells were processed for confocal microscopy, using anti-lamin A/C antibodies and Hoechst 33342 to visualize, respectively, the nuclear lamina and nuclear DNA. Arrows indicate transfected cells, with their nuclei shown in a 3D reconstruction at right. Scale bar, 50 μm.

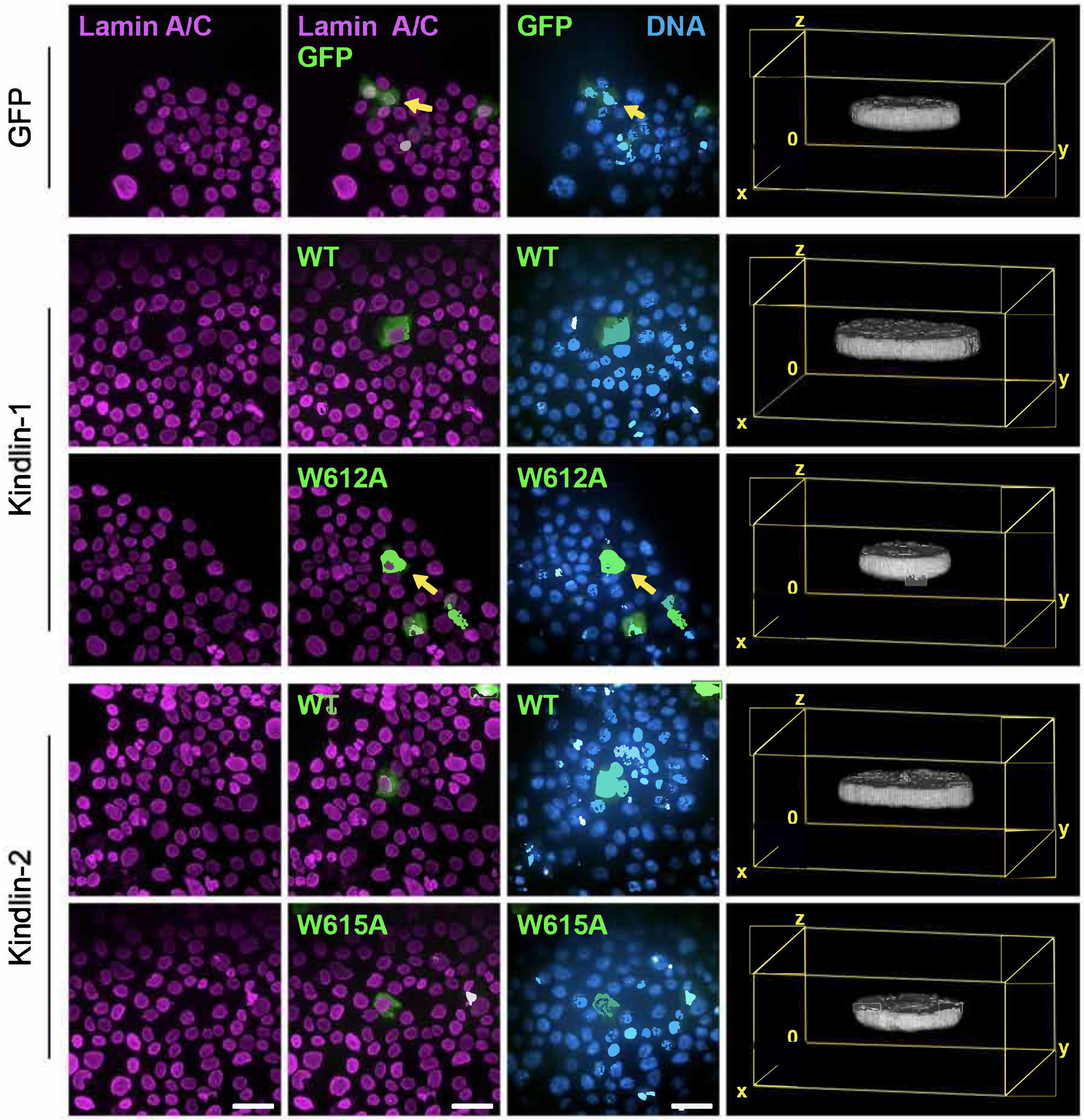

Figure S8. Effect of exogenous Kindlin proteins on nuclear dimensions in SCC13 KO2 cells. Cells were transfected with vectors encoding GFP or the indicated FLAG-tagged Kindlin proteins.Twenty-eight hours after transfection, the cells were processed for confocal microscopy, using anti-lamin A/C antibodies and Hoechst 33342 to visualize, respectively, the nuclear lamina and nuclear DNA. Arrows indicate transfected cells, with their nuclei shown in a 3D reconstruction at right. Scale bar, 50 μm.

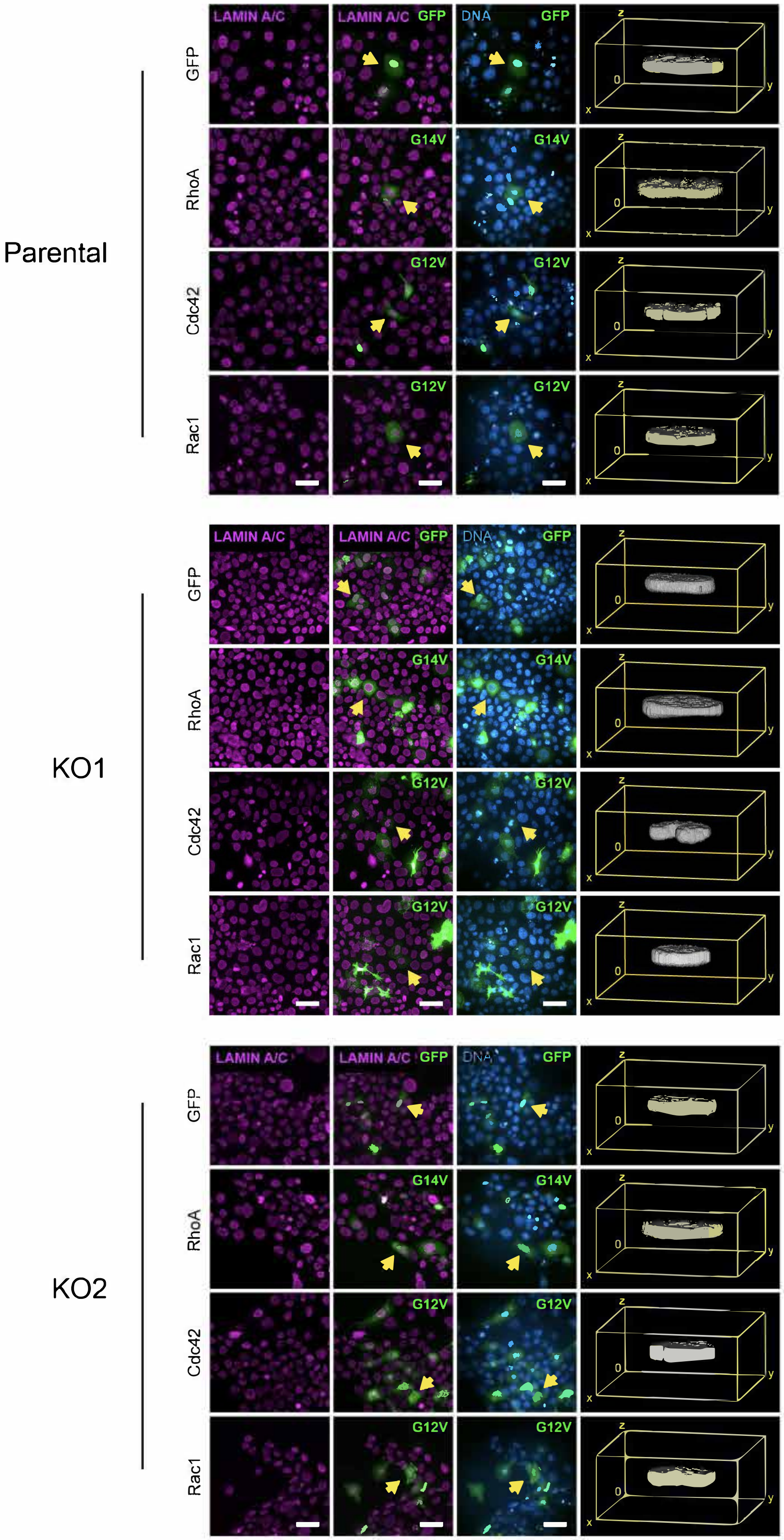

Figure S9. Effect of constitutively active GTPase proteins on nuclear dimensions in parental, KO1 and KO2 cells. Cells were transfected with vectors encoding GFP or the indicated GFP-tagged proteins.Twenty-eight hours after transfection, the cells were processed for confocal microscopy, using anti-lamin A/C antibodies and Hoechst 33342 to visualize,respectively, the nuclear lamina and nuclear DNA. Arrows show transfected GFP-positive cells, with their nuclei shown in a 3D reconstruction at right. Scale bar, 50 μm.

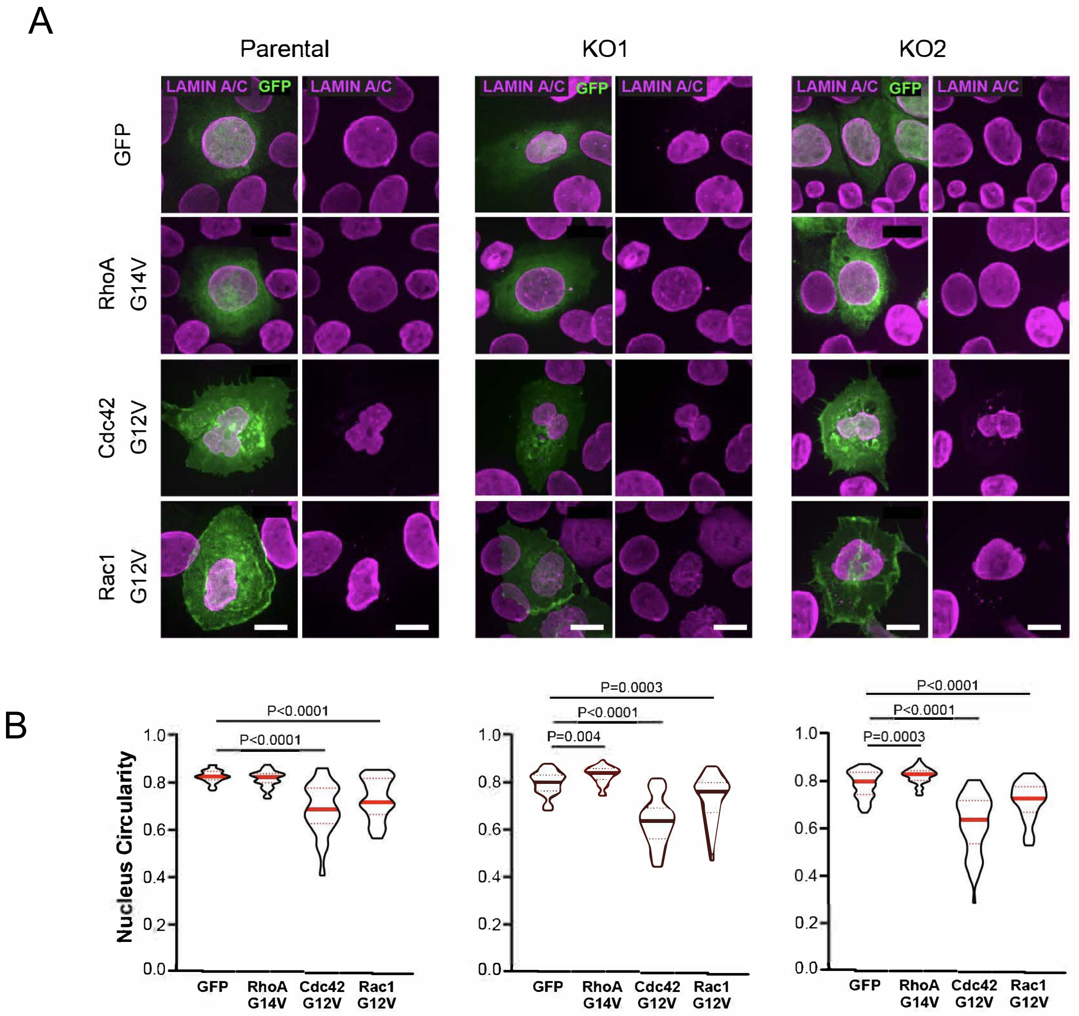

Figure S10. Effect of constitutively active Rho GTPases on nuclear morphology in SCC13 cells. (A) Parental, KO1 and KO2 cells were transfected with vectors encoding the indicated GFP-tagged Rho GTPase proteins. Cells were processed for con-focal microscopy 28 h after transfection. Transfected cells were identified from maximum intensity projections by direct GFP fluorescence, and the nuclear lamina was visualized with anti-lamin A/C antibodies. Scale bar, 25 μm. (B) Violin plots show median, 75th and 25th percentile circularity values. P values were determined using a mixed effects model, with Dunnet's post-hoc test for multiple comparisons against GFP-transfected cells (n=3; 11-26 nuclei scored for each biological replicate).

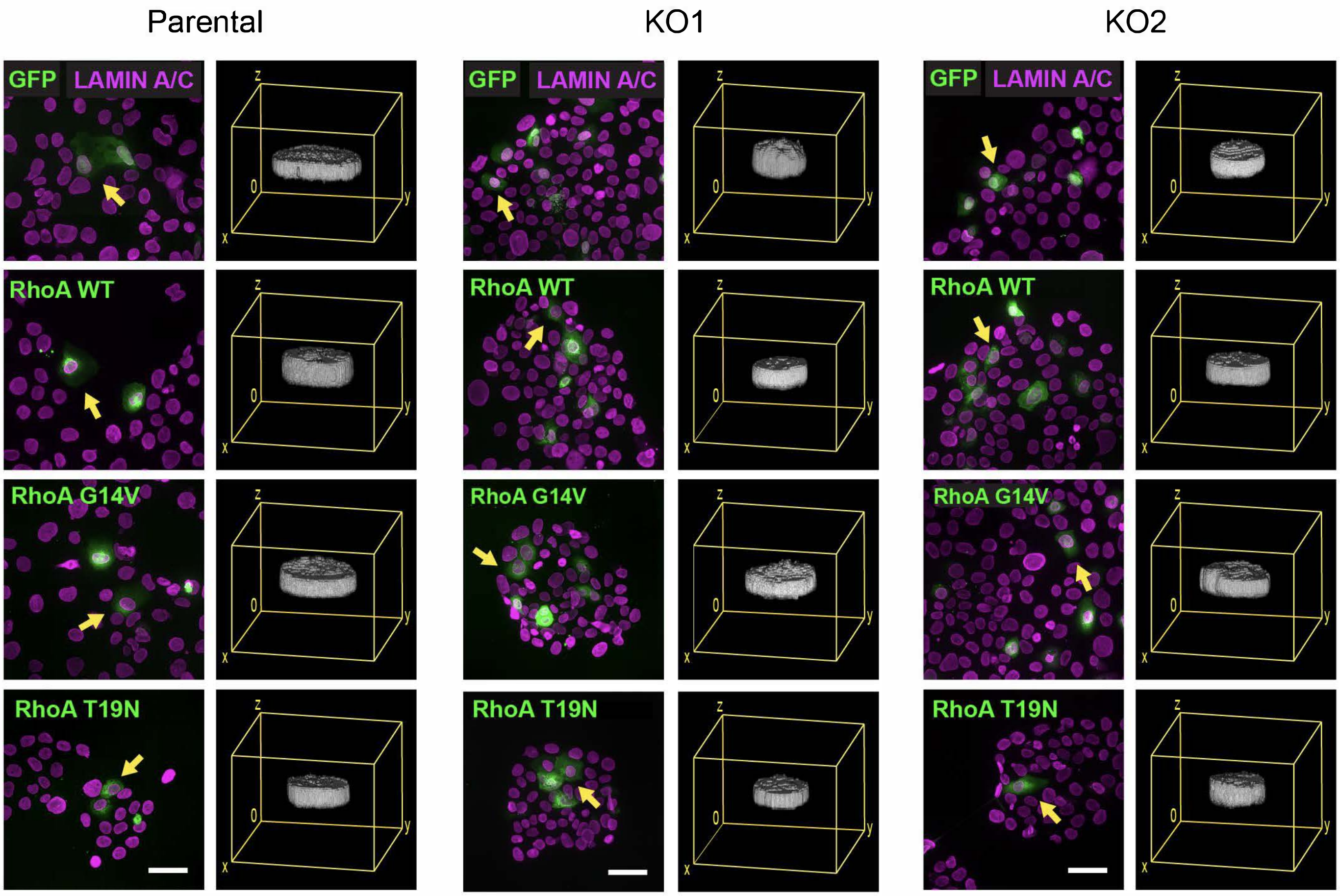

Figure S11. Effect of RhoA GTPase proteins on nuclear dimensions in Parental, KO1 and KO2 cells. Cells were transfected with vectors encoding GFP or the indicatedGFP-tagged RhoA proteins.Twenty-eight hours after transfection, the cells were processed for confocal microscopy, using anti-lamin A/C antibodies to visualize the nuclear lamina. Transfected cells were identified by direct GFP fluorescence. Arrows indicate the transfected GFP-positive cells whose corresponding nuclei are shown in a 3D reconstruction at right. Scale bar, 50 μm.

Supplemental Methods

Measurement of 2-dimensional cell area from confocal z-stack images

Parental, KO1 and KO2 SCC13 cells were transfected with a vector encoding green fluorescent protein (GFP). Twenty-eight hours after transfections, the cells were processed for confocal microscopy. Z-stacks were acquired from GFP-positive cells that were identified by direct fluorescence. Two-dimensional area of GFP-expressing transfected cells was estimated from maximum-intensity projections (MIP) of the z-stacks acquired. To this end, the maximum-intensity projections of GFP-positive cells generated were segmented with the Otsu method using Fiji 2.16.0/1.54p software. The cell area from these projections was estimated from individual cell outlines. A total of 30 cells for each cell line were scored (10 cells/biological replicate; 3 biological replicates). The graph below shows data points presented as area measured in those individual transfected cells that were located at the periphery of a cell cluster (P) or that were located in a central position of a cell cluster (C) and, thus, were surrounded by multiple untransfected cells. Area measurements were analyzed with GraphPad Prism v8.02, using a mixed-effects model within a repeated measures Two-way ANOVA, with Tukey's post-hoc test. Significance was set at $P<0.05$. Gray, blue and orange data points represent values from a given individual biological replicate.

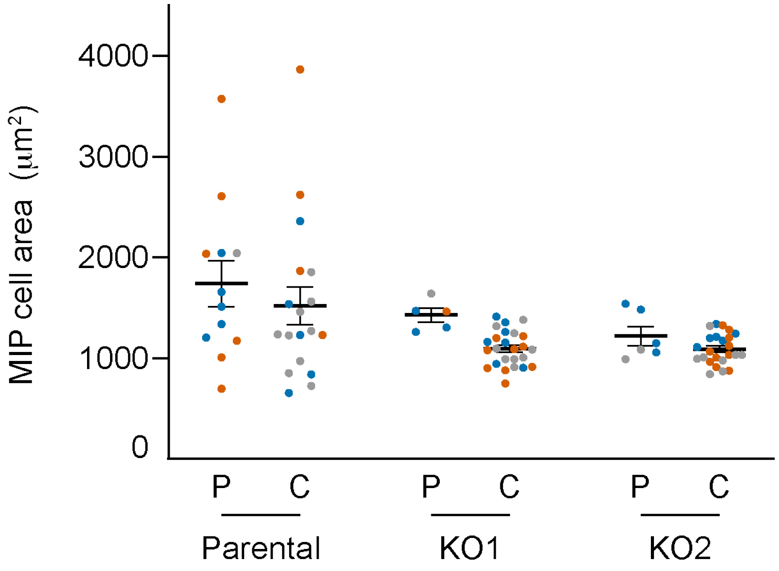

Supplement: Supplementary file 1 [file cells-15-01991-s001.zip › cells-4574469-supplementary.pdf]
